# TET2 as a tumor suppressor and therapeutic target in T-cell acute lymphoblastic leukemia

**DOI:** 10.1073/pnas.2110758118

**Published:** 2021-08-19

**Authors:** Maike Bensberg, Olof Rundquist, Aida Selimović, Cathrine Lagerwall, Mikael Benson, Mika Gustafsson, Hartmut Vogt, Antonio Lentini, Colm E. Nestor

**Affiliations:** ^a^Crown Princess Victoria Children’s Hospital, Department of Biomedical and Clinical Sciences, Linköping University, 581 83 Linköping, Sweden;; ^b^Bioinformatics, Department of Physics, Chemistry and Biology, Linköping University, 581 83 Linköping, Sweden;; ^c^Department of Medical Biochemistry and Biophysics, Karolinska Institute, 171 77 Stockholm, Sweden

**Keywords:** TET2, T-ALL, 5-azacytidine, HERV, vitamin C

## Abstract

Pediatric T-cell acute lymphoblastic leukemia (T-ALL) is an aggressive malignancy resulting from overproduction of immature T-cells in the thymus and is typified by widespread alterations in DNA methylation. As survival rates for relapsed T-ALL remain dismal (10 to 25%), development of targeted therapies to prevent relapse is key to improving prognosis. Whereas mutations in the DNA demethylating enzyme TET2 are frequent in adult T-cell malignancies, *TET2* mutations in T-ALL are rare. Here, we analyzed RNA-sequencing data of 321 primary T-ALLs, 20 T-ALL cell lines, and 25 normal human tissues, revealing that *TET2* is transcriptionally repressed or silenced in 71% and 17% of T-ALL, respectively. Furthermore, we show that *TET2* silencing is often associated with hypermethylation of the *TET2* promoter in primary T-ALL. Importantly, treatment with the DNA demethylating agent, 5-azacytidine (5-aza), was significantly more toxic to *TET2*-silenced T-ALL cells and resulted in stable re-expression of the *TET2* gene. Additionally, 5-aza led to up-regulation of methylated genes and human endogenous retroviruses (HERVs), which was further enhanced by the addition of physiological levels of vitamin C, a potent enhancer of TET activity. Together, our results clearly identify 5-aza as a potential targeted therapy for *TET2*-silenced T-ALL.

## Significance

Pediatric T-cell acute lymphoblastic leukemia (T-ALL) is an aggressive malignancy in need of novel targeted therapies to prevent relapse and lessen treatment toxicity. We reveal frequent (∼88%) transcriptional silencing or repression of the tumor suppressor *TET2* in T-ALL. We show that loss of *TET2* in T-ALL is correlated with hypermethylation of the *TET2* promoter and that *TET2* expression can be rescued by treatment with the DNA demethylating agent, 5-azacytidine (5-aza). We further reveal that the TET2 cofactor vitamin C exerts a strong synergistic effect on global transcriptional changes when added to 5-aza treatment. Importantly, 5-aza treatment results in increased cell death, specifically in T-ALL cells lacking TET2. Thus, we clearly identify 5-aza as a potentially targeted therapy for *TET2*-silenced T-ALL.

Aberrant DNA methylation is a common feature of cancer, typified by locus-specific gains of methylation at gene promoters and genome-wide loss of DNA methylation in intergenic regions. Loss-of-function mutations in enzymes involved in the homeostasis of DNA methylation are frequent across a range of malignancies (1), and DNA-demethylating drugs have been successfully used in treatment of myeloid malignancies (2–5), supporting a role for DNA methylation in tumorigenesis and its clinical relevance as a target for treatment.

The TET methylcytosine dioxygenases (TET1-3) initiate DNA demethylation by conversion of 5-methylcystosine to 5-hydroxymethylcytosine (5hmC) (6–8). *TET2* mutations are particularly common in hematopoietic malignancies including acute myeloid leukemia (23%), myelodysplastic syndromes (25%), chronic myelomonocytic leukemia (50%), and peripheral T-cell lymphomas (60%) (1). In addition, loss of TET2 function can be phenocopied by gain-of-function mutations in the isocitrate dehydrogenase genes (*IDH1* and *IDH2*), resulting in production of 2-hydroxygluterate (2-HG), a potent inhibitor of TET2 activity (9, 10). In adult T-cell leukemia/lymphoma (ATLL), reduced genomic 5hmC is associated with a more acute, aggressive form of the malignancy and worse prognosis. Loss of 5hmC in ATLL was shown to be independent of mutations in *TET2* but was instead caused by reduced *TET2* expression (11), indicating that TET2 function can be inhibited by mutation, 2-HG production or altered transcription. In support of a tumor suppressive role for TET2 in hematopoiesis, mice lacking *Tet2* display an array of hematopoietic abnormalities including enhanced stem cell renewal, myeloproliferation, decreased common lymphoid progenitors, and a predisposition to development of hematological malignancies (12–14). In humans, *TET2* mutations are frequently associated with age-related clonal hematopoiesis (15), and children carrying autosomal recessive mutations in *TET2* exhibit skewed T-cell development, altered B-cell maturation, and a pronounced susceptibility for development of lymphomas (16). Analogous to the use of DNA demethylating agents, therapeutic interventions that enhance TET2 enzymatic activity, decrease 2-HG inhibition, or restore *TET2* transcription may be clinically beneficial in hematological malignancies. Indeed, a growing number of studies have reported that vitamin C treatment rescued altered 5hmC levels in *TET2*-mutated cancers, directly reducing tumorigenic potential of cancer cells (17, 18), rendering malignant cells more sensitive to standard anticancer agents (19, 20) or by inducing an innate immune response through up-regulation of human endogenous retroviruses (HERVs) (21, 22). Furthermore, a recent study reported overexpression of *TET1* in T-cell acute lymphoblastic leukemia (T-ALL) and TET1-mediated promotion of leukemic growth to be dependent upon its catalytic ability to produce 5hmC (23), supporting the hypothesis that the DNA demethylation pathway represents an avenue for therapeutic action.

Pediatric T-ALL is an aggressive malignancy caused by failure of normal T-cell maturation in the thymus. Like all pediatric malignancies, T-ALL has a low mutational burden, but nonetheless, pediatric T-cell malignancies must overcome the same barriers to transformation as clearly facilitated by TET2 loss in adult T-cell malignancies. Increasing intensity of chemotherapy has improved prognosis of T-ALL from 50% to 75% between 1970 and 2000 (24). However, the last 20 y have seen only marginal improvements in outcome, with survival rates for relapsed T-ALL remaining just 10% to 25% (25). In addition, the long and intensive course of chemotherapy employed in T-ALL frequently results in serious, often fatal, side effects (26). Thus, a key clinical challenge to increasing survival in T-ALL is development of targeted therapies to prevent relapse and decrease toxicity of treatment (25, 26).

Here, we investigated the expression of the *TET2* gene in healthy human progenitor T cells, pediatric T-ALL patients, and T-ALL cell lines, revealing that *TET2* was frequently silenced in T-ALL and that silencing was associated with aberrant hypermethylation of the *TET2* promoter. Furthermore, we found that treatment with the DNA demethylating agent, 5-azacytidine (5-aza), was significantly more toxic to *TET2*-silenced T-ALL cells and resulted in stable re-expression of the *TET2* gene. The 5-aza treatment was associated with up-regulation of both methylated genes and HERVs. Moreover, 5-aza–mediated transcriptional reprogramming was associated with *TET2* expression levels and was further enhanced by addition of physiological levels of vitamin C, a potent enhancer of TET2 activity. Together, our results reveal *TET2* as a potential tumor suppressor in T-ALL and a marker for response to 5-aza in this poorly understood malignancy.

## Results

### The Tumor Suppressor *TET2* Is Frequently Silenced in T-ALL.

**Fig. 1.**
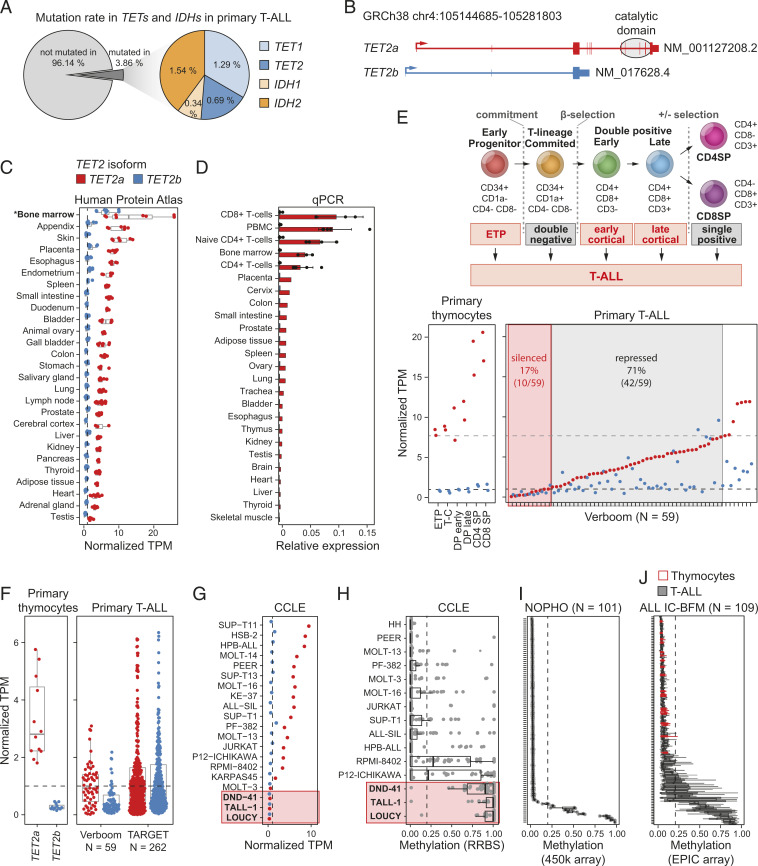
TET2 is a novel tumor suppressor in T-ALL. (*A*) Mutations in *TETs* and *IDHs* in primary human T-ALL from a meta-analysis of nine studies including 854 patients. (*B*) Known *TET2* isoforms, *TET2a* (red) and *TET2b* (blue). (*C*) Expression of *TET2* isoforms in 27 healthy tissues from the Human Protein Atlas. (*D*) Expression of *TET2* isoforms relative to housekeeping gene *GAPDH* in 25 human tissues. Data without error bars is based on RNA pools of three individuals each. (*E*, *Upper*) Schematic representation of human thymocyte development from ETP to CD4 or CD8 single positive T cells (CD4SP, CD8SP). (*Lower*) Expression of *TET2a* (red) and *TET2b* (blue) in the 6 human thymocyte subtypes (*Left*) and 59 primary T-ALL patients (*Right*). Dotted black line: silenced *TET2* (TPM < 1), gray dotted line: repressed *TET2* (one-sample *t* test, *P* < 0.05, compared to ETP, T-C, and DP primary thymoctes). T-C, T cell-committed; DP, double positive. (*F*) Expression of *TET2* in primary human thymocytes and T-ALL patients from two independent studies. (*G*) *TET2* expression in T-ALL cancer cell lines based on RNA-seq data from Broad Institute CCLE. (*H*–*J*) Methylation of the *TET2* promoter (*H*) in T-ALL cell lines based on RRBS from CCLE and in primary human T-ALL patients from (*I*) NOPHO and (*J*) the ALL IC-BFM protocol including 20 samples from healthy human thymocytes (red). (*C*, *F*, and *G*) Dotted black line at TPM = 1 as cutoff for silencing. (*H*–*J*) Dotted black line at methylation = 0.2.

Whereas mutations in *TET2* have been sporadically reported in T-ALL, no systematic analysis of *TET2* mutation frequency in T-ALL has been performed to date. Meta-analysis of targeted exome sequencing, whole exome sequencing, and whole genome sequencing of leukemic blasts from 854 T-ALL patients (Dataset S1) confirmed that *TET2* mutations were infrequent in T-ALL (0.7%) (Fig. 1*A* and *SI Appendix*, Fig. S1*A*). In addition, the paucity of *IDH1/2* mutations (1.9%) suggested that metabolic inhibition of TET2 by aberrant 2-HG production was also uncommon in T-ALL (Fig. 1*A*). Mutations in *TET2* or *IDHs* were similarly rare in 24 analyzed T-ALL cell lines (*SI Appendix*, Fig. S1*B*).

Since *TET2* has recently been reported to be frequently misexpressed in ATLL (11), we next investigated if *TET2* expression was similarly altered in T-ALL. To understand the potential consequence of any *TET2* expression change observed in T-ALL, we first needed to establish normal *TET2* expression patterns across human tissues. The *TET2* gene has two isoforms, a full-length transcript, *TET2a*, and a shorter transcript, *TET2b*, which lacks the C-terminal catalytic domain (Fig. 1*B*). By analyzing RNA-sequencing (RNA-seq) from 27 healthy tissues from the Human Protein Atlas (27), no expression of *TET2b* was detected in any tissue except bone marrow, in which expression of *TET2b* was considerably lower than *TET2a* (Fig. 1*C*). Additionally, qPCR with isoform-specific probes in an independent panel of 25 normal human tissues confirmed absence of *TET2b* expression in all tissues, including bone marrow (Fig. 1*D*). Our results cast doubt on the validity of *TET2b* as a genuine transcript in humans, the annotation of which was based on a single sequence observed in a single fetal kidney complementary DNA (cDNA) library (28). Indeed, *TET2b*-specific exonic sequences showed no conservation across vertebrates and a lack of mapped reads in seven fetal RNA-seq libraries, including kidney. Furthermore, analysis of splicing revealed no splice junctions over *TET2b*-specific exon 1 and exon 2 (*SI Appendix*, Fig. S1*C* and Dataset S2) (29). In contrast, *TET2a* was expressed in all tissues with highest expression in hematopoietic cells (Fig. 1 *C* and *D*), establishing *TET2a* as the main and potentially only *TET2* transcript in humans.

T-ALL can arise from any stage of T-cell development from early thymic progenitors (ETP) to differentiated single positive T cells (Fig. 1*E*, *Upper*). Revealing aberrant gene expression in T-ALL has been hampered by a lack of gene expression data for the normal progenitor T-cell (thymocyte) populations from which T-ALL derives. Using recently published RNA-seq data for the six canonical thymocyte subpopulations in humans (30), we could confirm that *TET2* was highly expressed in all normal postnatal thymocyte populations isolated from children undergoing cardiac surgery (30). In contrast, compared to ETP, T-cell–committed primary thymocytes, and double positive primary thymocytes, *TET2* expression was repressed (one-sample *t* test, *P* < 0.05) or silenced (transcript per million [TPM] < 1) in 71% (42/59) and 17% (10/59) of primary pediatric T-ALL, respectively (Fig. 1*E*, *Lower*) (31). A similar loss of *TET2* expression was also observed in RNA-seq data of leukemic blasts from an independent cohort of 262 pediatric T-ALL patients (Fig. 1*F*) (32). Whereas *TET2* and *TET3* are known to have overlapping enzymatic function (1, 33), loss of *TET2* was not associated with up-regulation of *TET3* in T-ALL (*SI Appendix*, Fig. S1*E*). Thus, *TET2* expression is aberrantly down-regulated or absent in the majority of pediatric T-ALL.

Our observations in primary T-ALL were supported by analysis of RNA-seq data for 20 T-ALL cell lines from the Cancer Cell Line Encyclopedia (CCLE) (34), which showed a similar distribution of *TET2* down-regulation and silencing (Fig. 1*G*). Interestingly, reduced representation bisulfite sequencing (RRBS) data for 15 of the same T-ALL cell lines (34) revealed that the *TET2* promoter was hypermethylated in all but one cell line in which *TET2* was silent (Fig. 1*H*) and showed a tendency toward higher methylation in cell lines in which *TET2* expression was reduced (Fig. 1*H* and *SI Appendix*, Fig. S1*D*). Importantly, whole genome DNA methylation data for two independent cohorts (*N*_NOPHO_ = 101, *N*_ALL_ _IC-BFM_ = 109) of pediatric T-ALL patients (30, 35, 36) revealed hypermethylation of the *TET2* promoter in a subset of patients (9/101 Nordic Society for Pediatric Hematology and Oncology [NOPHO]; 8/109 International Berlin-Frankfurt-Munster Study Group trials 2009 [ALL IC-BFM]) (Fig. 1 *I* and *J*), suggesting one mechanism of *TET2* silencing may be through aberrant DNA methylation. As with most molecular lesions in T-ALL, loss of *TET2* did not associate with molecular subtype, prognosis, or key clinical parameters in a well-characterized cohort of 262 pediatric T-ALL patients (*SI Appendix*, Fig. S1*F*) (32).

Together, the tumor suppressor gene (TSG) *TET2* is frequently silenced in T-ALL and may be a driver of T-ALL transformation and potential therapeutic target.

### Vitamin C Toxicity in T-ALL Is Independent of TET Activity.

**Fig. 2.**
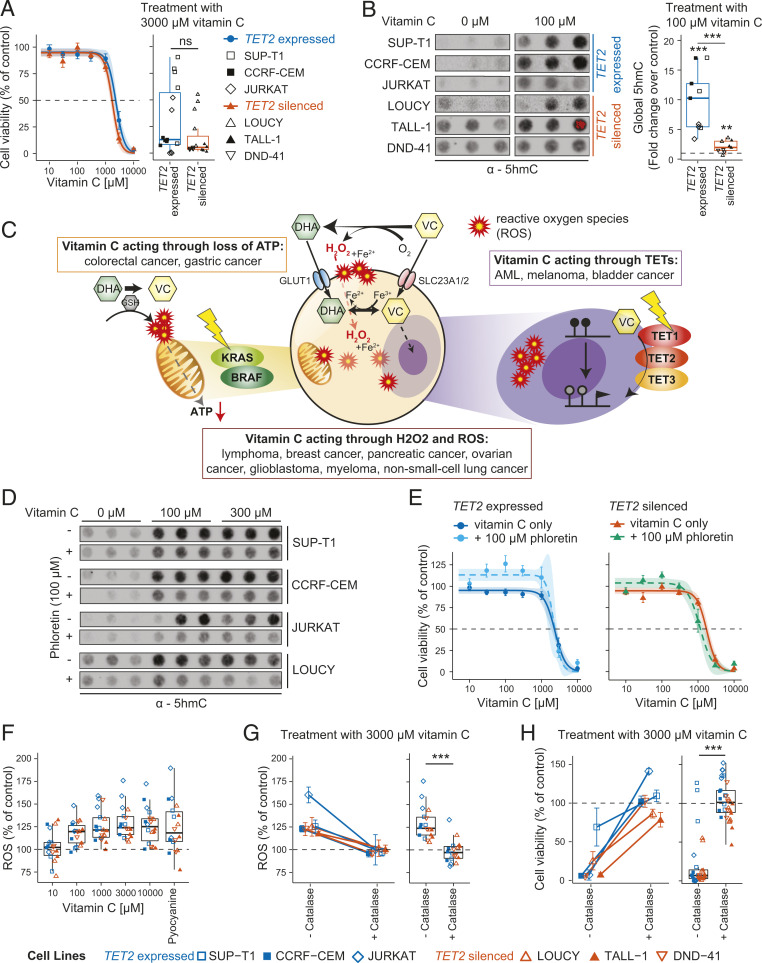
Cytotoxicity of vitamin C (VC) is caused by ROS independently of TET activity in vitro. (*A*) Cell viability curve for T-ALL cell lines with expressed and silenced *TET2* treated with increasing concentrations of VC for 24 h (*Left*) and boxplot showing cell viability for treatment with 3,000 μM VC compared to untreated control (*Right*). (*B*) Dot blot using an antibody against 5hmC in T-ALL cell lines treated with a H_2_O control or 100 μM VC for 24 h in triplicates (*Left*); quantification of signaling intensity comparing cell lines with expressed and silenced *TET2* relative to untreated control samples (dotted line) (*Right*). (*C*) Treatment with VC can mediate its cytotoxic effect through three main pathways: 1) production of hydrogen peroxide (H_2_O_2_) and ROS during oxidation of VC to DHA, 2) energy crisis in cancers with a *KRAS* or *BRAF* mutation (yellow flash) after DHA is reduced back to VC intracellularly, or 3) cofactor of the TET enzymes (TET1-3) leading to increased enzymatic activity. GSH, glutathione. (*D*) Dot blot showing global levels of 5hmC in T-ALL cell lines after treatment for 24 h with H_2_O control or indicated concentrations of VC in the absence (−) or presence (+) of 100 μM phloretin. (*E*) Cell viability curves for T-ALL cell lines treated with increasing concentrations of VC with or without 100 μM phloretin for cell lines with expressed *TET2* (*Left*) or silenced *TET2* (*Right*). Biological triplicates for three different cell lines each (n_expressed_ = 9 and n_silenced_ = 9). (*F*) Boxplot showing levels of total (intracellular and extracellular) ROS created in response to treatment of T-ALL cell lines with VC at increasing concentrations relative to an untreated control (dotted line); pyocyanine used as a positive control. (*G*) Intracellular and extracellular ROS created in response to treatment of T-ALL cell lines with 3,000 μM VC without and with the addition of catalase for each cell line individually (*Left*) and box plot representing all cell lines (*Right*) relative to an untreated control (dotted line). (*H*) Cell viability of T-ALL cell lines after treatment with 3,000 μM VC without and with the addition of catalase for each cell line separately (*Left*) and all cell lines combined (*Right*). Percentage of untreated control. (*A*, *B*, *G*, and *H*) Two-tailed, unpaired Student’s *t* test; ns, *P* > 0.05; *P ≤ 0.05; **P ≤ 0.01; ***P ≤ 0.001.

Having revealed frequent repression or loss of *TET2* expression in T-ALL, we next sought to test if vitamin C, a potent enhancer of TET activity, could rescue loss of TET2 by increasing the catalytic activity of the remaining TET enzymes. Indeed, decreased levels of global 5hmC are associated with worse prognosis in ATLL (11), and TET2 and TET3 have been shown to have redundant functions in both hematopoiesis and leukemogenesis (1, 33). To dissect the potential of vitamin C as a targeted therapy in T-ALL with loss of TET2, we obtained six T-ALL cell lines in which *TET2* was expressed and unmethylated (SUP-T1, CCRF-CEM, JURKAT) or silent and methylated (LOUCY, TALL-1, DND-41) (Fig. 1 *G* and *H* and *SI Appendix*, Fig. S1*B*). Expression status of all three *TET* genes and global 5hmC levels were assessed by qPCR and immuno-dot blot, respectively (*SI Appendix*, Fig. S2 *A* and *B*), showing no difference in *TET1* and *TET3* expression nor in 5hmC levels relative to *TET2* status. Treatment of these cell lines with increasing vitamin C concentrations spanning physiological (10 to 100 μM) and pharmacological (300 to 10,000 μM) concentrations revealed similar cytotoxicity in all cell lines, albeit with a tendency toward increased toxicity in *TET2*-deficient cells (not significant) (Fig. 2*A* and *SI Appendix*, Fig. S2*C*). Furthermore, treatment with physiological vitamin C (100 μM) resulted in a pronounced increase in global 5hmC levels at 24 h, with a lower relative increase observed in TET2-deficient cells (Fig. 2*B*). Notably, a similar relative increase in global 5hmC levels was achieved with just 10 μM vitamin C as with elevated physiological concentrations (100 and 300 μM) (*SI Appendix*, Fig. S2 *D* and *E*), neither of which concentration was cytotoxic to any T-ALL cell line. The 5hmC levels at higher concentrations could not be reliably assessed due to cell death (*SI Appendix*, Fig. S2*F*). Importantly, increased 5hmC levels were not associated with increased expression of any *TET* gene (*SI Appendix*, Fig. S2*G*). Thus, supplementation of T-ALL cells with vitamin C at concentrations obtainable with oral administration (100 μM) resulted in a 2 to 10-fold increase in 5hmC levels but did not affect cell viability.

The observation that treatment with physiological vitamin C concentrations (100 μM) resulted in increased global 5hmC levels in T-ALL cells in the absence of toxicity suggested that vitamin C toxicity was not mediated by the TET enzymes (17, 18, 37, 38) (Fig. 2 *A* and *B*). Vitamin C, however, can elicit toxicity through several pathways other than enhancement of TET activity (Fig. 2*C*). Having confirmed expression of the main vitamin C (*SLC23A1* and *SLC23A2*) and dehydroascorbic acid (DHA; *GLUT1*) transporters in each T-ALL cell line (*SI Appendix*, Fig. S3*A*), we blocked transport through SLC23A1, SLC23A2, and GLUTs with the inhibitor phloretin (39, 40). As expected, pharmacological inhibition of vitamin C uptake resulted in reduced 5hmC production compared with vitamin C alone (Fig. 2*D* and *SI Appendix*, Fig. S3 *B* and *C*). However, inhibition of vitamin C uptake did not reduce toxicity in T-ALL cell lines irrespective of *TET2* status (Fig. 2*E* and *SI Appendix*, Fig. S3*D*).

The finding that vitamin C toxicity did not require its transport across the cell membrane, ruling out cytotoxicity through increased TET activity and DHA mediated loss of ATP (41, 42), suggested extracellular production of reactive oxygen species (ROS) as the potential source of cytotoxicity (Fig. 2*C*) (43, 44). Indeed, addition of vitamin C to culture medium resulted in increased levels of intracellular (*SI Appendix*, Fig. S4*A*) and total (intracellular + extracellular) ROS (Fig. 2*F* and *SI Appendix*, Fig. S4*B*), while supplementation with catalase, which decomposes H_2_O_2_ to H_2_O and O_2_, maintained ROS at background levels, even at high (3,000 μM) concentrations of vitamin C (Fig. 2*G*). Importantly, catalase completely rescued vitamin C–induced cytotoxicity in T-ALL cell lines without affecting the enhancing activity on 5hmC production (Fig. 2*H* and *SI Appendix*, Fig. S4*C*). Indeed, treatment with H_2_O_2_ confirmed its ability to induce cytotoxicity in three T-ALL cell lines independent of *TET2* expression (SUP-T1, JURKAT) or silencing (LOUCY) (*SI Appendix*, Fig. S4*D*).

Thus, cytotoxicity of vitamin C observed in T-ALL cell lines in vitro is completely independent of its stimulatory effect on TET enzymatic activity.

### 5-Aza Induces *TET2* Expression and Targets *TET2*-Silenced Cells.

**Fig. 3.**
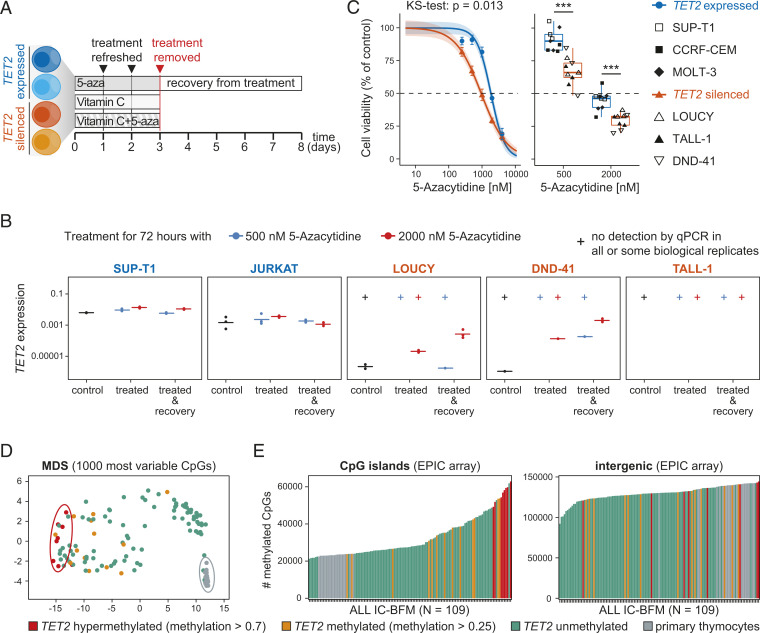
5-Aza induces *TET2* expression and targets *TET2*-silenced cells. (*A*) Schematic representation of experimental setup. T-ALL cell lines with silenced or expressed *TET2* were treated with 5-aza, vitamin C, or a combination of both for 3 d followed by removal of treatment and a recovery period of 5 d. (*B*) Expression (qPCR) of *TET2* relative to housekeeping gene *GAPDH* in T-ALL cell lines with expressed (SUP-T1, JURKAT) and silenced (LOUCY, DND-41, TALL-1) *TET2* after treatment with 500 nM or 2,000 nM 5-aza for 72 h or after treatment followed by recovery without treatment for 5 d. No Ct value in one or more than one biological replicate indicated as no detection by qPCR (+). (*C*) Cell viability curve for T-ALL cell lines with wild-type (3 cell lines) and silenced (3 cell lines) *TET2* treated with increasing concentrations of 5-aza for 72 h, comparison by Kolmogorov-Smirnov test (*Left*). Boxplot showing cell viability for treatment with 500 and 2,000 nM 5-aza compared to untreated control; two-tailed, unpaired Student’s *t* test; ns, *P* > 0.05; *P ≤ 0.05; **P ≤ 0.01; ***P ≤ 0.001 (*Right*). *n* = 3 for each cell line. (*D*) Multidimensional scaling (MDS) plot including the 1,000 most variable CpGs for the ALL IC-BFM 2009 protocol cohort including healthy primary thymocytes (EPIC array). (*E*) Number of methylated CpGs in CpG islands (*Left*) and intergenic regions (*Right*) in the ALL IC-BFM 2009 cohort including healthy primary thymocytes (EPIC array). (*D* and *E*) Status of *TET2* promoter methylation indicated by color.

Given our observation that *TET2* silencing in primary and cultured T-ALL cells was associated with *TET2* promoter methylation (Fig. 1 *G* and *H*), we next tested the ability of the Food and Drug Administration–approved DNA demethylating agent, 5-aza, to rescue *TET2* gene expression in T-ALL as an alternative therapeutic approach. Five T-ALL cell lines with normal (SUP-1, JURKAT) and silenced (LOUCY, DND-41, TALL-1) *TET2* expression were treated with 5-aza for 72 h followed by a recovery period without treatment for 5 d (Fig. 3*A*). We were able to successfully induce expression of positive control genes *DAZL* and *GAGE* in all cell lines except for TALL-1 at 5-aza concentrations showing low cytotoxicity, 500 nM (10% to 23% toxicity), and high cytotoxicity, 2,000 nM (54% to 70% toxicity) (*SI Appendix*, Fig. S5*A*). We detected expression of *TET2* in DND-41 cells after treatment with both concentrations of 5-aza and in LOUCY for 2,000 nM while *TET2* expression remained stable in SUP-T1 and JURKAT cells (Fig. 3*B*). Expression of *TET2* was further increased 5 d after withdrawal of 5-aza in LOUCY and DND-41 cell lines (Fig. 3*B*), suggesting a stable and heritable up-regulation of *TET2*. Lack of *TET2* up-regulation in TALL-1 cells likely reflects their significantly slower replication rate, and consequent incorporation of 5-aza compared with DND-41 and LOUCY cells as evidenced by the lack of up-regulation of 5-aza–sensitive control genes in TALL-1 cells (*SI Appendix*, Fig. S5*A*). Importantly, treatment of *TET2* wild-type (SUP-T1, CCRF-CEM, MOLT-3) and *TET2*-deficient (LOUCY, TALL-1, DND-41) cell lines with 5-aza also resulted in significantly higher cytotoxicity in cells lacking *TET2* (Kolmogorov-Smirnov test, *P* = 0.013) (Fig. 3*C* and *SI Appendix*, Fig. S5*B*). Toxicity of 5-aza to TALL-1 cells (Fig. 3*C*) in the absence of *TET2* up-regulation (Fig. 3*B*) shows that toxicity is not mediated by TET2 directly but that *TET2* methylation is a marker of 5-aza sensitivity in T-ALL. Loss of *TET2* compared to healthy thymocytes may result in the establishment of a distinct methylation profile that renders cells particularly sensitive to treatment with a global DNA-demethylating agent. Indeed, analysis of global methylation profiles in primary T-ALL cells revealed that *TET2*-silenced T-ALL cells have a distinct global methylation pattern (Fig. 3*D*) marked by extreme promoter hypermethylation but also have a tendency toward increased methylation of intergenic cytosine-guanine-dinucleotides (CpGs) (Fig. 3*E*), contrary to the intergenic hypomethylation observed in most malignancies (45), including T-ALL. A highly similar clustering by DNA methylation profile was also observed in *TET2*-deficient T-ALL cell lines (*SI Appendix*, Fig. S5*C*). Together, our findings suggest that a TET2-associated hypermethylation phenotype may render T-ALL cells more sensitive to treatment with DNA demethylating agents, independent of reactivation of TET2 itself.

### 5-Aza and Vitamin C Act Synergistically to Increase Expression of Methylated Genes and Endogenous Retroviruses.

**Fig. 4.**
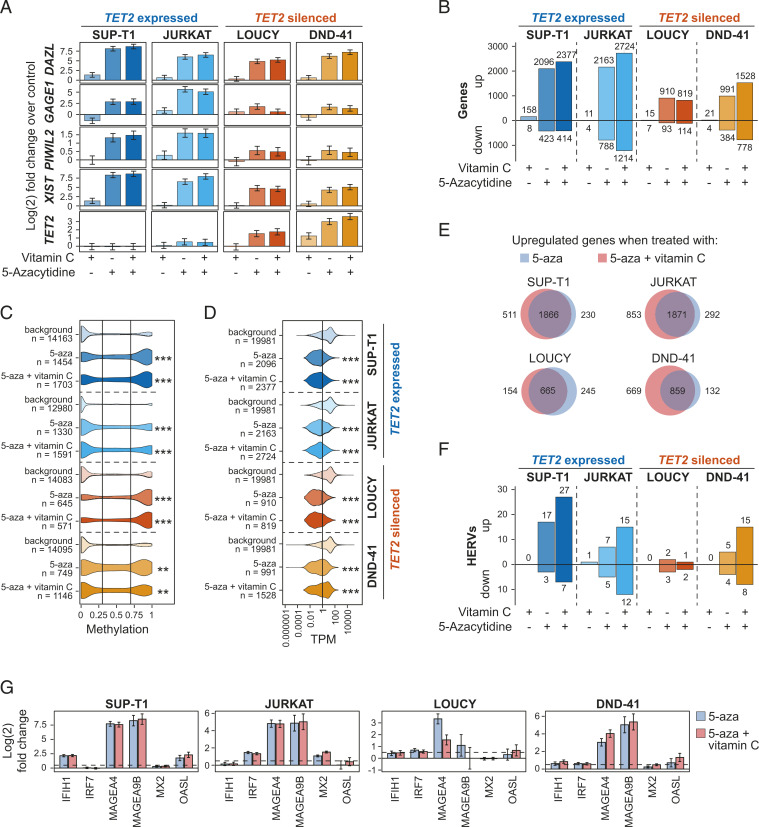
5-Aza and vitamin C act synergistically to increase expression of methylated genes and HERVs. (*A*) Expression of *TET2* and selected methylated genes in response to treatment with 2,000 μM 5-aza and 100 μM vitamin C for 72 h in T-ALL cell lines with expressed (SUP-T1, JURKAT) and silenced (LOUCY, DND-41) *TET2*. Differential expression based on total RNA sequencing of biological duplicates shown as log (2) fold change relative to an untreated control. (*B*) Number of differentially expressed genes in *TET2*-expressing and *TET2*-deficient cells when treated with 2,000 μM 5-aza and 100 μM vitamin C for 72 h. (*C*) Base-resolution promoter DNA methylation in untreated T-ALL cell lines for genes that become up-regulated following treatment with 2,000 μM 5-aza and 100 μM vitamin C for 72 h. (*D*) Expression (TPM) in untreated T-ALL cell lines for genes that become up-regulated following treatment with 2,000 μM 5-aza and 100 μM vitamin C for 72 h. (*E*) Venn diagrams showing overlap of significantly up-regulated genes [log (2) fold change > 0.5, p_adjusted_ < 0.05] in four T-ALL cell lines treated with 2,000 μM 5-aza only or 5-aza plus 100 μM vitamin C. (*F*) Number of differentially expressed HERVs [log (2) fold change > 0.5, p_adjusted_ < 0.05] in *TET2*-expressing and *TET2*-deficient cells when treated with 2,000 μM 5-aza and 100 μM vitamin C for 72 h. (*G*) Expression of interferon type I and antiviral response genes in response to treatment with 2,000 μM 5-aza with and without 100 μM vitamin C for 72 h in T-ALL cell lines. Expression shown as log (2) fold change of biological duplicates relative to an untreated control. Dotted line shows log (2) fold change of 0.5. (*C* and *D*) Fisher’s exact test, Benjamini-Hochberg, FDR-corrected *P*; ns, *P* > 0.05; *P ≤ 0.05; **P ≤ 0.01; ***P ≤ 0.001.

To shed light on the potential mechanisms of 5-aza sensitivity in *TET2*-silenced cells, we next sought to identify the transcriptional changes induced by 5-aza in T-ALL cell lines. Based on our observation of *TET2* up-regulation in response to 5-aza (Fig. 3*B*) and the pronounced effect of vitamin C on TET activity (Fig. 2*B*), we additionally aimed to investigate vitamin C as a potential supplement to treatment with 5-aza. To this end, we treated two cell lines with silenced and methylated *TET2* (LOUCY, DND-41) and two cell lines expressing *TET2* (SUP-T1, JURKAT) with 2,000 nM 5-aza, 100 μM vitamin C, or a combination of both for 3 d followed by total RNA sequencing (Fig. 3*A*). A minimum of 66 million read pairs of 150 bp each allowed for analysis of both coding and noncoding RNA (Dataset S3). The 5-aza induced expression of genes known to be silenced by DNA methylation (*DAZL*, *GAGE1*, *PIWIL2*, and *XIST*) (46, 47) as well as *TET2* as determined by total RNA-seq (Fig. 4*A*) and qPCR (*SI Appendix*, Fig. S6*A*). As expected, treatment with 5-aza led to a global increase in gene expression in all cell lines (Fig. 4*B*) with up-regulated genes being enriched for both promoter hypermethylation (methylation > 0.7) and no expression (TPM < 1) in untreated control samples (Fig. 4 *C* and *D* and Datasets S4 and S5). Sixty-three genes were exclusively up-regulated in both *TET2*-silenced cell lines, LOUCY and DND-41, but neither SUP-T1 nor JURKAT (*SI Appendix*, Fig. S6*B* and Dataset S6), among which were genes connected to promoter hypermethylation, tumorigenicity, and poor prognosis in a range of malignancies (48–52). As seen by others (53), up-regulated genes in response to 5-aza were not enriched for TSGs in any of the four cell lines analyzed (hypergeometric test; p_adjusted_ > 0.09 in all cell lines). Indeed, TSGs were significantly down-regulated by 5-aza treatment (Dataset S7). In stark contrast, we again observed up-regulation of *TET2*, an established TSG in numerous hematopoietic malignancies, in both LOUCY and DND-41 cells (Fig. 4*A* and *SI Appendix*, Fig. S6*A*), highlighting the exceptionality of *TET2* silencing and re-expression by DNA demethylating agent 5-aza in T-ALL.

Although vitamin C alone had little effect on gene expression (Fig. 4*B*), a pronounced global effect on gene expression was observed upon complementing 5-aza treatment with 100 μM vitamin C. Addition of vitamin C led to an increase in the number of up-regulated genes in all cell lines except LOUCY (Fig. 4*B*). Interestingly, LOUCY cells (Fig. 4*A*) displayed the lowest *TET2* expression of all four cell lines after 5-aza treatment (*SI Appendix*, Fig. S6*A*). Most genes up-regulated by 5-aza and vitamin C in a given T-ALL cell line were also up-regulated after treatment with 5-aza alone (Fig. 4*E*) and displayed a further increase in expression in response to addition of vitamin C in SUP-T1, JURKAT, and DND-41 but not in LOUCY cells (*SI Appendix*, Fig. S6*C*).

In addition, 5-aza may induce toxicity in cancer cells by induction of HERV expression, which in turn leads to cell death (53–55). HERVs are located in intergenic regions and tend to be highly methylated (45), which can be assumed to be specifically true for *TET2*-silenced T-ALL in light of the hypermethylation profile observed in intergenic regions (Fig. 3*D*). Therefore, we next examined changes in HERV expression after demethylating treatment with and without vitamin C. Indeed, we observed up-regulation of retroviruses (Fig. 4*F*) including HERVs previously reported to be activated in response to 5-aza such as *HERV-Fc1*, *LTR12E*, *MER70A*, and *MER48* (21). While vitamin C alone did not lead to a change in HERV expression, the addition of vitamin C to 5-aza treatment had a profound additive effect on global HERV up-regulation as previously found by others (21). Adding vitamin C to 5-aza treatment resulted not only in a higher number of differentially expressed HERVs (Fig. 4*F*) but also a general increase of HERV expression in all T-ALL cell lines except JURKAT (*SI Appendix*, Fig. S6*D*), which was even more pronounced than the effect observed for genes (Fig. 4*B* and *SI Appendix*, Fig. S6*C*). Notably, LOUCY cells that had the weakest reactivation of *TET2* (Fig. 4*A*) were the only cell line that did not show an increased number of up-regulated HERVs (Fig. 4*F*) in response to supplementing 5-aza treatment with vitamin C.

Other studies have shown treatment with hypomethylating agents such as 5-aza and the resulting up-regulation of HERVs to lead to an interferon type I response (21, 53–56) with the strongest reaction seen to occur as many as 24 d after treatment (55). Even though our analysis is limited by a comparatively short timeframe, we observed increased expression of selected genes involved in double-stranded RNA sensing (*IFIH1*, *IRF7*), antiviral response (*MX2*, *OASL*), and antigen presentation (*MAGEA9B*, *MAGEA4*) (Fig. 4*G*) after only 3 d of treatment. While the addition of vitamin C to 5-aza resulted in a clear increase in differentially expressed genes and HERVs, it had no effect on toxicity in any cell line, independently of expression of *TET2* (*SI Appendix*, Fig. S6*E*).

Taken together, a physiologically relevant concentration of vitamin C had a strong synergistic effect in combination with 5-aza on both genes and HERV expression.

## Discussion

T-ALL patients are typically treated with a regime of intense chemotherapy. This approach often fails to prevent relapse and can result in high levels of treatment-related toxicity and death (57). Thus, a key challenge to improving prognosis in T-ALL is identification and targeting of novel pathways involved in pathogenesis and relapse. Here, we show that expression of the TSG *TET2* is repressed or silenced in 71% and 17% of primary T-ALL, respectively. As *TET2* is highly expressed in all progenitor T-cell populations from which T-ALL arises, loss of *TET2* expression in T-ALL is aberrant and does not reflect expression in the cell of origin. The frequency of *TET2* repression or loss in T-ALL even exceeds the frequency (30% to 65%) of *TET2* loss-of-function mutations observed in angioimmunoblastic T-cell lymphoma in adults (58–60). *TET2* mutations are common in blood cells in the general adult population and appear to confer an increased risk for development of hematopoietic malignancies as opposed to acting as the genetic lesion driving transformation (15). It is tempting to speculate that in pediatric malignancies, which have a substantially lower (5 to 10 fold) mutational burden than adults (61, 62), increased risk of transformation may be more readily conferred by alteration of TSG or oncogene expression than through genetic lesions.

As with all dioxygenases, the activity of the TET enzymes can be enhanced with vitamin C (63, 64) by facilitating rapid recycling of molecular iron required for TET catalytic activity (63, 65). The observation of overlapping and often redundant function of TET2 and TET3 (1, 33, 66) in murine T-cell development suggested that enhancement of TET3 activity in TET2-deficient cells may mimic TET2 recovery (18). Moreover, vitamin C resulted in reduced tumorigenic potential of bone marrow–derived cells from *tet2*^−/−^ mice but not cells from *tet2*^−/−^*tet3*^−/−^ double knockouts (18), again supporting a redundant function for Tet2 and Tet3 in hematopoiesis (1). Indeed, aberrant hematopoietic stem cell (HSC) renewal observed in a *tet2*^−/−^ mouse model was rescued by vitamin C treatment and closely mimicked genetic restoration of Tet2 (18). Similarly, vitamin C was shown to maintain normal Tet2 activity in HSCs in vivo, thereby decreasing HSC turnover and malignant transformation (17). Here, we observed both toxicity and an increase in 5hmC production in six T-ALL cell lines upon addition of vitamin C. However, 5hmC production was independent of toxicity in vitro, which was due to production of extracellular ROS. These results are in line with early studies on vitamin C toxicity in a range of cancer cell lines (43, 44) but may fail to capture more complex cytotoxic effects of vitamin C that may occur in vivo. Any direct cytotoxic effects of increased 5hmC production in response to vitamin C treatment may require long-term treatment regimens to allow the increased 5hmC to result in replication-dependent DNA demethylation and subsequent transcriptional reprogramming.

As *TET2* silencing was typically associated with hypermethylation of its promoter region, we next explored the possibility of restoring *TET2* expression using the nonspecific DNA demethylating agent 5-aza. Indeed, 5-aza treatment resulted in robust and stable re-expression of *TET2* in two of three *TET2*-silenced T-ALL cell lines (LOUCY and DND-41) even after removal of 5-aza, supporting a role for DNA methylation in *TET2* suppression. This is a critical finding as we have previously shown that most promoter hypermethylation in cancer occurred at genes that 1) were already silent in the tissue from which the cancer derived (67) and 2) consequently were not re-expressed upon treatment with 5-aza (68). Here, our findings suggest that DNA-demethylating agents can rescue aberrant silencing of the key tumor suppressor, TET2 in T-ALL cells. Importantly, 5-aza was significantly more toxic to all T-ALL cell lines in which *TET2* was silenced. However, re-expression of *TET2* was not required to elicit increased toxicity in all cell lines (TALL-1), suggesting that DNA methylation alterations associated with loss of TET2 may render cells more sensitive to the DNA demethylating effects of 5-aza. Indeed, T-ALL cell lines lacking *TET2* showed a distinct hypermethylation phenotype, which was also observed in primary T-ALL cells.

Total RNA-seq of 5-aza–treated cells confirmed up-regulation of methylated genes in all cell lines, including *TET2* in LOUCY and DND-41 cells. A general enrichment of apoptotic pathways was not observed, consistent with the highly pleiotropic effect of 5-aza. However, several well-characterized apoptosis-related (e.g., DNA damage sensing) genes were specifically up-regulated in *TET2*-silenced cells. For instance, *DDIAS*, a known suppressor of apoptosis in response to DNA damage (69), was induced by 5-aza treatment exclusively in LOUCY and DND-41 but not in *TET2*-expressing cells. Upon closer inspection, *DDIAS* was highly expressed in untreated SUP-T1 and JURKAT while being silenced in TET2-deficient cells. The lack of expression of *DDIAS* might play a role in sensitizing *TET2*-silenced cells to treatment with 5-aza, which induces DNA damage.

As expected, treatment of T-ALL cell lines with a physiologically relevant concentration of vitamin C (100 μM) resulted in little change in global gene or HERV expression. However, treatment with 100 μM vitamin C and 5-aza in combination resulted in a marked synergistic effect. Moreover, the synergistic effect of vitamin C was most notable in DND-41 cells, in which *TET2* re-expression had been most pronounced, suggesting an additive effect of 5-aza–mediated DNA demethylation and TET2-mediated DNA demethylation. Alternatively, reduction of global DNA methylation levels by 5-aza may render the T-ALL epigenome more responsive to the enhancing effect of vitamin C on histone demethylases, since vitamin C has been shown to act as a cofactor for several Jumonji-catalytic-domain–containing histone demethylases such as KDM2A-B (H3K36me), KDM3A (H3K9me), KDM4A-E (H3K36me and H3K9me), and KDM6A-B (H3K27me) (70). A similarly pronounced synergistic effect was observed in HERV expression, with a clear association between *TET2* re-expression and treatment effect size. Whereas further experiments are required to determine the molecular underpinnings of 5-aza toxicity in *TET2*-silenced T-ALL cells, our results clearly identify TET2 as a potential tumor suppressor and, importantly, a therapeutic target in T-ALL.

## Materials and Methods

### Meta-Analysis for Mutations in Primary T-ALL.

Data for mutations in T-ALL from whole exome sequencing, whole genome sequencing, or targeted exome sequencing were collected and summarized from nine recently published studies (Dataset S1), resulting in a dataset including 584 patients.

### Mutations in T-ALL Cell Lines.

Information on mutations in T-ALL cell lines was collected from Broad Institute’s CCLE, and the functional impact of missense mutations was assessed using the PROVEAN Web Server Tool (71) and the mutation assessor online tool (72). A consensus on the functional impact of a missense mutation was reached based on the PROVEAN and SIFT score (both from PROVEAN tool) and the combined functional score from mutation assessor.

### RNA-Seq Transcriptome Quantification from Publicly Available Data.

Transcriptome mapping was performed using Salmon v0.12.0 (–gcBias–seqBias–numBootstraps 100–validateMappings) (73) using known Refseq transcripts (NM_* and NR_*) from the GRCh38.p12 assembly (GCF_000001405.38_GRCh38.p12_rna.fna.gz) as a reference. Library type options were Human Protein Atlas (27): IU, TARGET T-ALL: ISR, CCLE: ISR. We did not observe any effect of adapter or quality trimming on abundance estimates in our data and analyzed fastq files directly. Abundance estimates were converted to h5 format using Wasabi (https://github.com/COMBINE-lab/wasabi), and data normalization and differential expression analysis was performed using likelihood ratio tests and gene-level *P* value aggregation as implemented in Sleuth (74, 75). Fetal RNA-seq samples from GSE111930 were aligned using STAR v2.6.0c (76). Conservation phylop100way scores of the TET2 genomic region (±5 kb) was acquired from the University of California Santa Cruz (UCSC) genome browser. RNA-seq read coverage was visualized with Gviz (77) in R, and splice junctions and conservation were added with IGV (78).

### 450k and EPIC Methylation Array Analysis.

Pediatric T-ALL data from NOPHO (35) was acquired and preprocessed using functional normalization (79) as implemented in R/minfi (80), and a general probe mask was applied to filter out bad probes (81). *TET2* promoter location was defined as Refseq NM_001127208.2 TSS extended 1 kb upstream and downstream.

### Cell Culture.

All cell lines were kept in a humidified incubator at 37 °C with 5% CO_2_ in American Type Culture Collection–modified RPMI (Roswell Park Memorial Institute) medium 1640 (Gibco Thermo Fisher Scientific, A10491) supplemented with 10% fetal bovine serum (Gibco Thermo Fisher Scientific, 10500) and 1% penicillin-streptomycin (100 U/mL penicillin, 100 μg/mL streptomycin) (Gibco Thermo Fisher Scientific, 15140). Cells were passaged every 2 to 3 d as necessary and regularly checked for mycoplasma (Lonza, LT07-318) to ensure no mycoplasma contamination was present.

### Cell Culture Reagents and Treatment.

Vitamin C (Sigma-Aldrich, A4544) was diluted in distilled H_2_O and pH was adjusted to 7 by addition of NaOH. To create a stock of 500 mM, 500 mg vitamin C was dissolved in 2.975 mL distilled H_2_O and 2.7 mL 1M NaOH. Aliquots for single use were kept at −20 °C. Catalase (Sigma-Aldrich, C1345) was dissolved directly in RPMI medium and used at a concentration of 600 U/mL. Phloretin (ACROS Organics, 307651000) was dissolved in DMSO and stored at −20 °C. 5-Aza (Sigma-Aldrich, A1287) was dissolved in RPMI medium, and aliquots were stored at −20 °C short term for up to 2 mo. The 1 × 10^5^ cells (for vitamin C, phloretin, and catalase treatment) or 1.5 × 10^5^ cells (for treatment with 5-aza) were treated in 500 μL compound diluted in RPMI medium at 37 °C for 24 h (vitamin C, phloretin, catalase) or 72 h (5-aza, 5-aza plus vitamin C) with refreshment of treatment after 24 and 48 h. Treatments with distilled H_2_O and DMSO were used as controls for vitamin C and phloretin treatment, respectively.

### Isolation and Sorting of Primary Immune Cells.

Peripheral blood mononuclear cells were isolated from buffy coats by healthy donors using Lymphoprep (Axis-Shield) and further separated based on CD4 with magnetic beads (CD4+ T-cell isolation kit, Miltenyi Biotec, 130-096-533). The CD4-positive fraction was used as-is, and from the CD4 negative fraction, CD8+ cells were enriched by staining with fluorescently labeled antibodies against CD3+ (Anti-CD3-Facific Blue, clone UCHT1, BioLegend, 300418) and CD8+ (Anti-CD8-APC, clone RPA-T8, BD, 555369) followed by fluorescence-activated cell sorting. Naïve CD4+ T-cells were isolated with the help of a Naïve CD4 T-Cell Isolation Kit (Miltenyi Biotec, 130-096-533).

### DNA and RNA Extraction.

DNA and RNA was extracted using the Quick-DNA/RNA Kit (Zymo Research, D7001) according to the manufacturer’s instructions. Nucleic acid concentration was determined by nanodrop.

### Conversion of RNA to cDNA and qPCR.

The 200 ng (cell lines) or 100 ng (primary tissues) RNA was treated with dsDNase I (Thermo Fisher, EN0771) for 2 min at 37 °C and converted into cDNA using the High Capacity cDNA Reverse Transcription Kit (Applied Biosystems, 4368814) at 37 °C for 2 h followed by inactivation for 5 min at 85 °C. cDNA was diluted to a volume of 200 μL, of which 5 μL was used for qPCR. qPCR was carried out in technical triplicates or duplicates (5-aza–treated cell lines) with the help of Fast Universal PCR Master Mix (Thermo Fisher Scientific, 4352042) using TaqMan probes (Thermo Fisher Scientific), as indicated in Dataset S8. qPCR was performed on a 7900HT Fast Real-Time PCR System (Applied Biosystems) with the following temperature profile: initial denaturation at 95 °C for 20 s and 40 cycles of 95 °C for 1 s and 60 °C for 20 s. Data were analyzed using the ΔCt or ΔΔCt method as appropriate with *GAPDH* as housekeeping gene. For analysis of *TET2* expression in human tissues 3 (CD8+ T-cells) or 5 (peripheral blood mononuclear cells, naïve CD4+ T-cell, CD4+ T-cells), biological replicates were analyzed. Additionally, *TET2* expression was assessed in three samples of commercially available bone marrow (biochain, ATR1234024; Zyagen, HR-704; TaKaRa, 636591) and a panel of 20 tissues (Ambion, AM6000), including pooled samples from three individuals each.

### Immuno-Dot Blot.

One microgram of DNA was diluted to a total volume of 200 μL in 400 mM NaOH and 10 mM EDTA and denatured at 95 °C for 15 min before blotting onto a positively charged nylon membrane (GE Healthcare, RPN119B) using vacuum suction (hybridization manifold, Harvard Apparatus). Membranes were washed shortly in 2× saline-sodium citrate (SSC) buffer (300 mM NaCl, 30 mM sodium citrate) and ultraviolet cross-linked for 1 min before baking at 80 °C for at least 2 h. Following this, membranes were blocked with 0.5% casein (Thermo Fisher Scientific, 37528) in TBS (20 mM Tris base, 150 mM NaCl) for 15 min and incubated with primary antibody against 5hmC (Active Motif, 39791; diluted 1:3,000 in blocking solution) for 1 h on ice. After three washes in TBS-Tween (0.1%), membranes were incubated with horseradish peroxidase (HRP)-conjugated secondary antibody (1:3,000, Bio-Rad, 1706515) for 1 h at room temperature. Membranes were washed two times in TBS-Tween and once in TBS before visualizing with the help of Clarity ECL Western Substrates (Bio-Rad, 1705060) and the ChemiDoc MP system (Bio-Rad). Quantification of signals for 5hmC was done using the image-processing software ImageJ (NIH). Antibody specificity was confirmed by blotting 10 ng of DNA standards with unmodified, methylated, or hydroxymethylated cytosine (GeneTex, GTX400004) with each blot.

### Alamar Blue Cell Viability Assay.

After treatment as described above, cells were washed twice with PBS to remove vitamin C and taken up in 500 μL 10% alamar blue cell viability reagent (Thermo Fisher Scientific, DAL1025) in RPMI medium. Following, 100 μL cell suspension per well was given into a 96-well plate in technical triplicates and incubated at 37 °C with 5% CO_2_ in a temperature and gas controlled plate reader (Tecan, Spark10M) for 12 h with absorbance at 570 nm and 600 nm being measured at the beginning and after 12 h. Analysis of reduced alamar blue, indicative of cell viability, relative to an untreated controls was carried out according the manual provided with the alamar blue assay solution. Cell viability curves were fitted to a 3 parameter logistic model using the *drc* R package (82).

### Annexin V and Propidium Iodide Staining and Flow Cytometry.

In a 12-well plate, 2 × 10^5^ cells were treated with vitamin C diluted in 1 mL RPMI medium for 24 h at standard incubator conditions (37 °C, 5% CO_2_). Following, cells were washed with Annexin V binding buffer (BioLegend, 422201) once and resuspended in 160 μL Annexin V binding buffer before staining with FITC-Annexin V (BioLegend, 421301) and Propidium iodide (BioLegend, 640906) for 15 min at room temperature in the dark. After diluting the cells by the addition of 500 μL Annexin V binding buffer, staining was analyzed using a Gallios Flow cytometer (Beckman Coulter). Compensation for spectral overlap of the two fluorophores was done using single stained control samples. Gating was based on single stained and unstained controls. All analyses of flow cytometry data were done utilizing the Kaluza analysis software (Beckman Coulter).

### ROS Detection.

#### Plate reader assay (detects extracellular and intracellular ROS).

The 2.5 × 10^4^ cells per well in a 96-well plate were treated with 100 μL of the desired concentration of vitamin C in ROS detection solution (Enzo, ENZ-51011) according to the manufacturer’s instructions. After 10 min of treatment, ROS was detected by measuring fluorescence (excitation: 488 nm, emission: 520 nm) in a Spark 10M plate reader (Tecan). Treatment with 500 μM Pyocyanine was used as a positive control for ROS creation.

#### Flow cytometry assay (detects intracellular ROS).

In a similar fashion as the plate reader assay, 1 × 10^5^ cells in a volume of 200 μL were treated with the desired concentration of vitamin C in flow cytometry tubes. After 30 min of treatment intracellular ROS was detected by fluorescence with a Gallios Flow Cytomoter (Beckman Coulter). Treatment with 500 μM Pyocyanine was used as a positive control for creation of ROS.

### RNA-Seq Library Preparation.

RNA for sequencing underwent in-column treatment with 5 U DNase I (Zymo Research, E1010) during RNA extraction. Only RNA samples with an RNA integrity number > 8 as shown by 2100 Bioanalyzer (Agilent) were chosen for RNA sequencing (average RIN score > 9.8).

The 400 ng of total RNA was rRNA depleted resulting in an rRNA content of <20%. rRNA depletion was followed by fragmentation, cDNA synthesis, end repair, adaptor ligation and PCR amplification. Pooled libraries were sequenced on the DNBSEQ sequencing platform with paired end reads of 150 bp and a sequencing depth of >60 million paired reads per sample.

### RNA-Seq Data Analysis.

#### Total RNA-seq expression analysis.

Transcriptome mapping was performed using Salmon v0.14.1 (–gcBias–seqBias–numBootstraps 100–validateMappings) (62) using known Refseq transcripts (NM_* and NR_*) from the GRCh38.p12 assembly (GCF_000001405.38_GRCh38.p12_rna.fna.gz) as a reference. Based on our findings (Fig. 1 *B*–*D* and *SI Appendix*, Fig. S1*C*) the *TET2* isoform *TET2b* (NM_017628) was excluded from the Salmon index generation. Abundance estimates were converted to h5 format using Wasabi (https://github.com/COMBINE-lab/wasabi) followed by data normalization and differential expression analysis performed using Wald tests implemented in Sleuth (63, 64). Differentially regulated genes were defined as qval < 0.05 (Wald test: BH, false discovery rate [FDR]-corrected *P*), with up-regulated and down-regulated defined as b (beta from sleuth) > 0.5 and b < −0.5 respectively. Overlap between cell line gene sets were explored using the VennDiagram (83) and UpSetR (84) packages in R.

#### DNA methylation analysis following treatment with 5-aza and vitamin C.

RRBS for T-ALL cell lines was acquired from Broad Institute’s CCLE (34). Promoter regions were defined as 1 kb upstream region of the TSS. Enrichment of methylated genes was tested using a Fisher’s exact test by comparing the ratio of methylted (CpG methylation > 0.7) genes and unmethylated genes (CpG methylation < 0.3) against the same ratio among all expressed genes.

#### Gene enrichment analysis.

Enrichment of TSGs and cancer testis antigens (CTA) was assayed by hypergeomtric tests, using “phyper,” in R. The TSG gene set was acquired from the NGC6.0 database (85) by selecting for all TSG marked genes and the CTA gene set from the CTdatabase (86).

#### HERV analysis.

To quantify expression of HERV families the RNA-seq samples were aligned using STAR v2.6.0c (76). The genomic position of all HERVs was acquired from the HERV database (69). Reads in HERVs were counted using “featurecounts” from the Rsubread package with the parameter "–primary" to only count primary alignments. The individual HERV counts were then loaded into R and summarized over 563 families using the match function. Differential HERV expression was then assessed using voom with LIMMA in R. A fold change of ±1.4 and an FDR ≤ 0.05 (two-tailed *t* test) was used to identify differentially expressed HERVs.

## Supplementary Material

Supplementary File

Supplementary File

Supplementary File

Supplementary File

Supplementary File

Supplementary File

Supplementary File

Supplementary File

Supplementary File

## Data Availability

The RNA-seq data reported in this paper have been deposited in the ArrayExpress database, https://www.ebi.ac.uk/arrayexpress/ (accession no. E-MTAB-10512) (87). All code for the RNA-seq analysis is available on github (https://gitlab.com/Olof_Rundquist/TET2_RNA_seq_analysis).

## References

[1] C. J. Lio, H. Yuita, A. Rao, Dysregulation of the TET family of epigenetic regulators in lymphoid and myeloid malignancies. Blood 134, 1487–1497 (2019). 31467060 10.1182/blood.2019791475 PMC6839946

[2] P. Fenaux.; International Vidaza High-Risk MDS Survival Study Group, Efficacy of azacitidine compared with that of conventional care regimens in the treatment of higher-risk myelodysplastic syndromes: A randomised, open-label, phase III study. Lancet Oncol. 10, 223–232 (2009). 19230772 10.1016/S1470-2045(09)70003-8 PMC4086808

[3] P. Fenaux., Azacitidine prolongs overall survival compared with conventional care regimens in elderly patients with low bone marrow blast count acute myeloid leukemia. J. Clin. Oncol. 28, 562–569 (2010). 20026804 10.1200/JCO.2009.23.8329

[4] J. J. Issa., Safety and tolerability of guadecitabine (SGI-110) in patients with myelodysplastic syndrome and acute myeloid leukaemia: A multicentre, randomised, dose-escalation phase 1 study. Lancet Oncol. 16, 1099–1110 (2015). 26296954 10.1016/S1470-2045(15)00038-8 PMC5557041

[5] S. C. Navada, J. Steinmann, M. Lübbert, L. R. Silverman, Clinical development of demethylating agents in hematology. J. Clin. Invest. 124, 40–46 (2014). 24382388 10.1172/JCI69739 PMC3871232

[6] S. Ito., Tet proteins can convert 5-methylcytosine to 5-formylcytosine and 5-carboxylcytosine. Science 333, 1300–1303 (2011). 21778364 10.1126/science.1210597 PMC3495246

[7] M. Tahiliani., Conversion of 5-methylcytosine to 5-hydroxymethylcytosine in mammalian DNA by MLL partner TET1. Science 324, 930–935 (2009). 19372391 10.1126/science.1170116 PMC2715015

[8] X. Wu, Y. Zhang, TET-mediated active DNA demethylation: Mechanism, function and beyond. Nat. Rev. Genet. 18, 517–534 (2017). 28555658 10.1038/nrg.2017.33

[9] L. Dang., Cancer-associated IDH1 mutations produce 2-hydroxyglutarate. Nature 462, 739–744 (2009). 19935646 10.1038/nature08617 PMC2818760

[10] J. A. Losman., (R)-2-hydroxyglutarate is sufficient to promote leukemogenesis and its effects are reversible. Science 339, 1621–1625 (2013). 23393090 10.1126/science.1231677 PMC3836459

[11] A. Marçais., Adult T cell leukemia aggressiveness correlates with loss of both 5-hydroxymethylcytosine and TET2 expression. Oncotarget 8, 52256–52268 (2016). 28881727 10.18632/oncotarget.13665 PMC5581026

[12] K. Moran-Crusio., Tet2 loss leads to increased hematopoietic stem cell self-renewal and myeloid transformation. Cancer Cell 20, 11–24 (2011). 21723200 10.1016/j.ccr.2011.06.001 PMC3194039

[13] E. Pronier., Inhibition of TET2-mediated conversion of 5-methylcytosine to 5-hydroxymethylcytosine disturbs erythroid and granulomonocytic differentiation of human hematopoietic progenitors. Blood 118, 2551–2555 (2011). 21734233 10.1182/blood-2010-12-324707 PMC3292425

[14] C. Quivoron., TET2 inactivation results in pleiotropic hematopoietic abnormalities in mouse and is a recurrent event during human lymphomagenesis. Cancer Cell 20, 25–38 (2011). 21723201 10.1016/j.ccr.2011.06.003

[15] S. Jaiswal., Age-related clonal hematopoiesis associated with adverse outcomes. N. Engl. J. Med. 371, 2488–2498 (2014). 25426837 10.1056/NEJMoa1408617 PMC4306669

[16] J. Stremenova Spegarova., Germline TET2 loss of function causes childhood immunodeficiency and lymphoma. Blood 136, 1055–1066 (2020). 32518946 10.1182/blood.2020005844

[17] M. Agathocleous., Ascorbate regulates haematopoietic stem cell function and leukaemogenesis. Nature 549, 476–481 (2017). 28825709 10.1038/nature23876 PMC5910063

[18] L. Cimmino., Restoration of TET2 function blocks aberrant self-renewal and leukemia progression. Cell 170, 1079–1095.e20 (2017). 28823558 10.1016/j.cell.2017.07.032 PMC5755977

[19] Y. Ma., High-dose parenteral ascorbate enhanced chemosensitivity of ovarian cancer and reduced toxicity of chemotherapy. Sci. Transl. Med. 6, 222ra18 (2014). 10.1126/scitranslmed.3007154 24500406

[20] A. Magrì., High-dose vitamin C enhances cancer immunotherapy. Sci. Transl. Med. 12, eaay8707 (2020). 32102933 10.1126/scitranslmed.aay8707

[21] M. Liu., Vitamin C increases viral mimicry induced by 5-aza-2′-deoxycytidine. Proc. Natl. Acad. Sci. U.S.A. 113, 10238–10244 (2016). 27573823 10.1073/pnas.1612262113 PMC5027469

[22] R. A. Luchtel., High-dose ascorbic acid synergizes with anti-PD1 in a lymphoma mouse model. Proc. Natl. Acad. Sci. U.S.A. 117, 1666–1677 (2020). 31911474 10.1073/pnas.1908158117 PMC6983418

[23] S. Bamezai., TET1 promotes growth of T-cell acute lymphoblastic leukemia and can be antagonized via PARP inhibition. Leukemia 35, 389–403 (2021). 32409690 10.1038/s41375-020-0864-3

[24] K. Schmiegelow.; Nordic Society of Paediatric Haematology and Oncology, Long-term results of NOPHO ALL-92 and ALL-2000 studies of childhood acute lymphoblastic leukemia. Leukemia 24, 345–354 (2010). 20010622 10.1038/leu.2009.251

[25] P. Quist-Paulsen., T-cell acute lymphoblastic leukemia in patients 1-45 years treated with the pediatric NOPHO ALL2008 protocol. Leukemia 34, 347–357 (2020). 31611626 10.1038/s41375-019-0598-2

[26] K. K. Ness, S. H. Armenian, N. Kadan-Lottick, J. G. Gurney, Adverse effects of treatment in childhood acute lymphoblastic leukemia: General overview and implications for long-term cardiac health. Expert Rev. Hematol. 4, 185–197 (2011). 21495928 10.1586/ehm.11.8 PMC3125981

[27] M. Uhlén., Proteomics. Tissue-based map of the human proteome. Science 347, 1260419 (2015). 25613900 10.1126/science.1260419

[28] S. Bechtel., The full-ORF clone resource of the German cDNA Consortium. BMC Genomics 8, 399 (2007). 17974005 10.1186/1471-2164-8-399 PMC2213676

[29] K. D. Wilson., Endogenous retrovirus-derived lncRNA BANCR promotes cardiomyocyte migration in humans and non-human primates. Dev. Cell 54, 694–709.e9 (2020). 32763147 10.1016/j.devcel.2020.07.006 PMC7529962

[30] J. Roels., Distinct and temporary-restricted epigenetic mechanisms regulate human αβ and γδ T cell development. Nat. Immunol. 21, 1280–1292 (2020). 32719521 10.1038/s41590-020-0747-9

[31] K. Verboom., A comprehensive inventory of TLX1 controlled long non-coding RNAs in T-cell acute lymphoblastic leukemia through polyA+ and total RNA sequencing. Haematologica 103, e585–e589 (2018). 29954933 10.3324/haematol.2018.190587 PMC6269303

[32] Y. Liu., The genomic landscape of pediatric and young adult T-lineage acute lymphoblastic leukemia. Nat. Genet. 49, 1211–1218 (2017). 28671688 10.1038/ng.3909 PMC5535770

[33] J. An., Acute loss of TET function results in aggressive myeloid cancer in mice. Nat. Commun. 6, 10071 (2015). 26607761 10.1038/ncomms10071 PMC4674670

[34] M. Ghandi., Next-generation characterization of the Cancer Cell Line Encyclopedia. Nature 569, 503–508 (2019). 31068700 10.1038/s41586-019-1186-3 PMC6697103

[35] J. Nordlund., Genome-wide signatures of differential DNA methylation in pediatric acute lymphoblastic leukemia. Genome Biol. 14, r105 (2013). 24063430 10.1186/gb-2013-14-9-r105 PMC4014804

[36] B. Szarzyńska-Zawadzka., PTEN abnormalities predict poor outcome in children with T-cell acute lymphoblastic leukemia treated according to ALL IC-BFM protocols. Am. J. Hematol. 94, E93–E96 (2019). 30614545 10.1002/ajh.25396

[37] C. B. Gustafson., Epigenetic reprogramming of melanoma cells by vitamin C treatment. Clin. Epigenetics 7, 51 (2015). 25977731 10.1186/s13148-015-0087-z PMC4430922

[38] D. Peng., Vitamin C increases 5-hydroxymethylcytosine level and inhibits the growth of bladder cancer. Clin. Epigenetics 10, 94 (2018). 30005692 10.1186/s13148-018-0527-7 PMC6045833

[39] J. M. May, The SLC23 family of ascorbate transporters: Ensuring that you get and keep your daily dose of vitamin C. Br. J. Pharmacol. 164, 1793–1801 (2011). 21418192 10.1111/j.1476-5381.2011.01350.x PMC3246704

[40] H. Tsukaguchi., A family of mammalian Na+-dependent L-ascorbic acid transporters. Nature 399, 70–75 (1999). 10331392 10.1038/19986

[41] J. Yun., Vitamin C selectively kills KRAS and BRAF mutant colorectal cancer cells by targeting GAPDH. Science 350, 1391–1396 (2015). 26541605 10.1126/science.aaa5004 PMC4778961

[42] Y. X. Lu., Pharmacological ascorbate suppresses growth of gastric cancer cells with GLUT1 overexpression and enhances the efficacy of oxaliplatin through redox modulation. Theranostics 8, 1312–1326 (2018). 29507622 10.7150/thno.21745 PMC5835938

[43] Q. Chen., Pharmacologic ascorbic acid concentrations selectively kill cancer cells: Action as a pro-drug to deliver hydrogen peroxide to tissues. Proc. Natl. Acad. Sci. U.S.A. 102, 13604–13609 (2005). 16157892 10.1073/pnas.0506390102 PMC1224653

[44] Q. Chen., Pharmacologic doses of ascorbate act as a prooxidant and decrease growth of aggressive tumor xenografts in mice. Proc. Natl. Acad. Sci. U.S.A. 105, 11105–11109 (2008). 18678913 10.1073/pnas.0804226105 PMC2516281

[45] M. Esteller, Epigenetics in cancer. N. Engl. J. Med. 358, 1148–1159 (2008). 18337604 10.1056/NEJMra072067

[46] J. A. Hackett., Promoter DNA methylation couples genome-defence mechanisms to epigenetic reprogramming in the mouse germline. Development 139, 3623–3632 (2012). 22949617 10.1242/dev.081661 PMC3436114

[47] X. Qiu., Equitoxic doses of 5-azacytidine and 5-aza-2'deoxycytidine induce diverse immediate and overlapping heritable changes in the transcriptome. PLoS One 5, e12994 (2010). 20927380 10.1371/journal.pone.0012994 PMC2947512

[48] T. L. Dunwell., Epigenetic analysis of childhood acute lymphoblastic leukemia. Epigenetics 4, 185–193 (2009). 19430199 10.4161/epi.4.3.8752

[49] Y. H. Hu., Hypermethylation of ADHFE1 promotes the proliferation of colorectal cancer cell via modulating cell cycle progression. OncoTargets Ther. 12, 8105–8115 (2019). 10.2147/OTT.S223423 PMC6782030 31632063

[50] A. Lasa., MEIS 1 expression is downregulated through promoter hypermethylation in AML1-ETO acute myeloid leukemias. Leukemia 18, 1231–1237 (2004). 15103390 10.1038/sj.leu.2403377

[51] K. Takane., Aberrant promoter methylation of PPP1R3C and EFHD1 in plasma of colorectal cancer patients. Cancer Med. 3, 1235–1245 (2014). 24861485 10.1002/cam4.273 PMC4302673

[52] A. K. Ward., Epigenetic silencing of CREB3L1 by DNA methylation is associated with high-grade metastatic breast cancers with poor prognosis and is prevalent in triple negative breast cancers. Breast Cancer Res. 18, 12 (2016). 26810754 10.1186/s13058-016-0672-x PMC4727399

[53] H. Ohtani., Activation of a subset of evolutionarily young transposable elements and innate immunity are linked to clinical responses to 5-azacytidine. Cancer Res. 80, 2441–2450 (2020). 32245794 10.1158/0008-5472.CAN-19-1696 PMC7507765

[54] K. B. Chiappinelli., Inhibiting DNA methylation causes an interferon response in cancer via dsRNA including endogenous retroviruses. Cell 162, 974–986 (2015). 26317466 10.1016/j.cell.2015.07.011 PMC4556003

[55] D. Roulois., DNA-demethylating agents target colorectal cancer cells by inducing viral mimicry by endogenous transcripts. Cell 162, 961–973 (2015). 26317465 10.1016/j.cell.2015.07.056 PMC4843502

[56] S. K. Saini., Human endogenous retroviruses form a reservoir of T cell targets in hematological cancers. Nat. Commun. 11, 5660 (2020). 33168830 10.1038/s41467-020-19464-8 PMC7653045

[57] N. Toft., Results of NOPHO ALL2008 treatment for patients aged 1-45 years with acute lymphoblastic leukemia. Leukemia 32, 606–615 (2018). 28819280 10.1038/leu.2017.265

[58] L. Couronné, C. Bastard, O. A. Bernard, TET2 and DNMT3A mutations in human T-cell lymphoma. N. Engl. J. Med. 366, 95–96 (2012). 22216861 10.1056/NEJMc1111708

[59] O. Odejide., A targeted mutational landscape of angioimmunoblastic T-cell lymphoma. Blood 123, 1293–1296 (2014). 24345752 10.1182/blood-2013-10-531509 PMC4260974

[60] F. Lemonnier., Recurrent TET2 mutations in peripheral T-cell lymphomas correlate with TFH-like features and adverse clinical parameters. Blood 120, 1466–1469 (2012). 22760778 10.1182/blood-2012-02-408542

[61] The ICGC/TCGA Pan-Cancer Analysis of Whole Genomes Consortium, Pan-cancer analysis of whole genomes. Nature 578, 82–93 (2020). 32025007 10.1038/s41586-020-1969-6 PMC7025898

[62] X. Ma., Pan-cancer genome and transcriptome analyses of 1,699 paediatric leukaemias and solid tumours. Nature 555, 371–376 (2018). 29489755 10.1038/nature25795 PMC5854542

[63] C. Kuiper, M. C. Vissers, Ascorbate as a co-factor for Fe- and 2-oxoglutarate dependent dioxygenases: Physiological activity in tumor growth and progression. Front. Oncol. 4, 359 (2014). 25540771 10.3389/fonc.2014.00359 PMC4261134

[64] J. I. Young, S. Züchner, G. Wang, Regulation of the epigenome by vitamin C. Annu. Rev. Nutr. 35, 545–564 (2015). 25974700 10.1146/annurev-nutr-071714-034228 PMC4506708

[65] K. M. Dickson, C. B. Gustafson, J. I. Young, S. Züchner, G. Wang, Ascorbate-induced generation of 5-hydroxymethylcytosine is unaffected by varying levels of iron and 2-oxoglutarate. Biochem. Biophys. Res. Commun. 439, 522–527 (2013). 24021282 10.1016/j.bbrc.2013.09.010 PMC4527183

[66] A. Tsagaratou., TET proteins regulate the lineage specification and TCR-mediated expansion of iNKT cells. Nat. Immunol. 18, 45–53 (2017). 27869820 10.1038/ni.3630 PMC5376256

[67] D. Sproul., Tissue of origin determines cancer-associated CpG island promoter hypermethylation patterns. Genome Biol. 13, R84 (2012). 23034185 10.1186/gb-2012-13-10-r84 PMC3491412

[68] D. Sproul., Transcriptionally repressed genes become aberrantly methylated and distinguish tumors of different lineages in breast cancer. Proc. Natl. Acad. Sci. U.S.A. 108, 4364–4369 (2011). 21368160 10.1073/pnas.1013224108 PMC3060255

[69] J. Y. Im., DNA damage-induced apoptosis suppressor (DDIAS), a novel target of NFATc1, is associated with cisplatin resistance in lung cancer. Biochim. Biophys. Acta 1863, 40–49 (2016). 26493727 10.1016/j.bbamcr.2015.10.011

[70] T. Lee Chong, E. L. Ahearn, L. Cimmino, Reprogramming the epigenome with vitamin C. Front. Cell Dev. Biol. 7, 128 (2019). 31380368 10.3389/fcell.2019.00128 PMC6646595

[71] Y. Choi, A. P. Chan, PROVEAN web server: A tool to predict the functional effect of amino acid substitutions and indels. Bioinformatics 31, 2745–2747 (2015). 25851949 10.1093/bioinformatics/btv195 PMC4528627

[72] B. Reva, Y. Antipin, C. Sander, Predicting the functional impact of protein mutations: Application to cancer genomics. Nucleic Acids Res. 39, e118 (2011). 21727090 10.1093/nar/gkr407 PMC3177186

[73] R. Patro, G. Duggal, M. I. Love, R. A. Irizarry, C. Kingsford, Salmon provides fast and bias-aware quantification of transcript expression. Nat. Methods 14, 417–419 (2017). 28263959 10.1038/nmeth.4197 PMC5600148

[74] H. Pimentel, N. L. Bray, S. Puente, P. Melsted, L. Pachter, Differential analysis of RNA-seq incorporating quantification uncertainty. Nat. Methods 14, 687–690 (2017). 28581496 10.1038/nmeth.4324

[75] L. Yi, H. Pimentel, N. L. Bray, L. Pachter, Gene-level differential analysis at transcript-level resolution. Genome Biol. 19, 53 (2018). 29650040 10.1186/s13059-018-1419-z PMC5896116

[76] A. Dobin., STAR: Ultrafast universal RNA-seq aligner. Bioinformatics 29, 15–21 (2013). 23104886 10.1093/bioinformatics/bts635 PMC3530905

[77] F. Hahne, R. Ivanek, Visualizing genomic data using Gviz and Bioconductor. Methods Mol. Biol. 1418, 335–351 (2016). 27008022 10.1007/978-1-4939-3578-9_16

[78] J. T. Robinson., Integrative genomics viewer. Nat. Biotechnol. 29, 24–26 (2011). 21221095 10.1038/nbt.1754 PMC3346182

[79] J. P. Fortin., Functional normalization of 450k methylation array data improves replication in large cancer studies. Genome Biol. 15, 503 (2014). 25599564 10.1186/s13059-014-0503-2 PMC4283580

[80] M. J. Aryee., Minfi: A flexible and comprehensive Bioconductor package for the analysis of Infinium DNA methylation microarrays. Bioinformatics 30, 1363–1369 (2014). 24478339 10.1093/bioinformatics/btu049 PMC4016708

[81] W. Zhou, P. W. Laird, H. Shen, Comprehensive characterization, annotation and innovative use of Infinium DNA methylation BeadChip probes. Nucleic Acids Res. 45, e22 (2017). 27924034 10.1093/nar/gkw967 PMC5389466

[82] C. Ritz, F. Baty, J. C. Streibig, D. Gerhard, Dose-response analysis using R. PLoS One 10, e0146021 (2015). 26717316 10.1371/journal.pone.0146021 PMC4696819

[83] H. Chen, P. C. Boutros, VennDiagram: A package for the generation of highly-customizable Venn and Euler diagrams in R. BMC Bioinformatics 12, 35 (2011). 21269502 10.1186/1471-2105-12-35 PMC3041657

[84] J. R. Conway, A. Lex, N. Gehlenborg, UpSetR: An R package for the visualization of intersecting sets and their properties. Bioinformatics 33, 2938–2940 (2017). 28645171 10.1093/bioinformatics/btx364 PMC5870712

[85] D. Repana., The Network of Cancer Genes (NCG): A comprehensive catalogue of known and candidate cancer genes from cancer sequencing screens. Genome Biol. 20, 1 (2019). 30606230 10.1186/s13059-018-1612-0 PMC6317252

[86] L. G. Almeida., CTdatabase: A knowledge-base of high-throughput and curated data on cancer-testis antigens. Nucleic Acids Res. 37, D816–D819 (2009). 18838390 10.1093/nar/gkn673 PMC2686577

[87] M. Bensberg, O. Rundquist, C. Nestor, RNA-seq of SUPT1, LOUCY, JURKAT and DND41 after treatment with 5-azacytidine and Vitamin C. EMB-EBI ArrayExpress. https://www.ebi.ac.uk/arrayexpress/experiments/E-MTAB-10512. Deposited 10 May 2021.

