# Supplementary material for: TET2 as a tumor suppressor and therapeutic target in T-cell acute lymphoblastic leukemia
Source: Proc Natl Acad Sci U S A. 2021 Aug 19;118(34):e2110758118. doi: 10.1073/pnas.2110758118 (PMC8403940; doi:10.1073/pnas.2110758118)
Supplement: Supplementary File [file pnas.2110758118.sapp.pdf]

Bensberg et al - Supplementary Figure 1

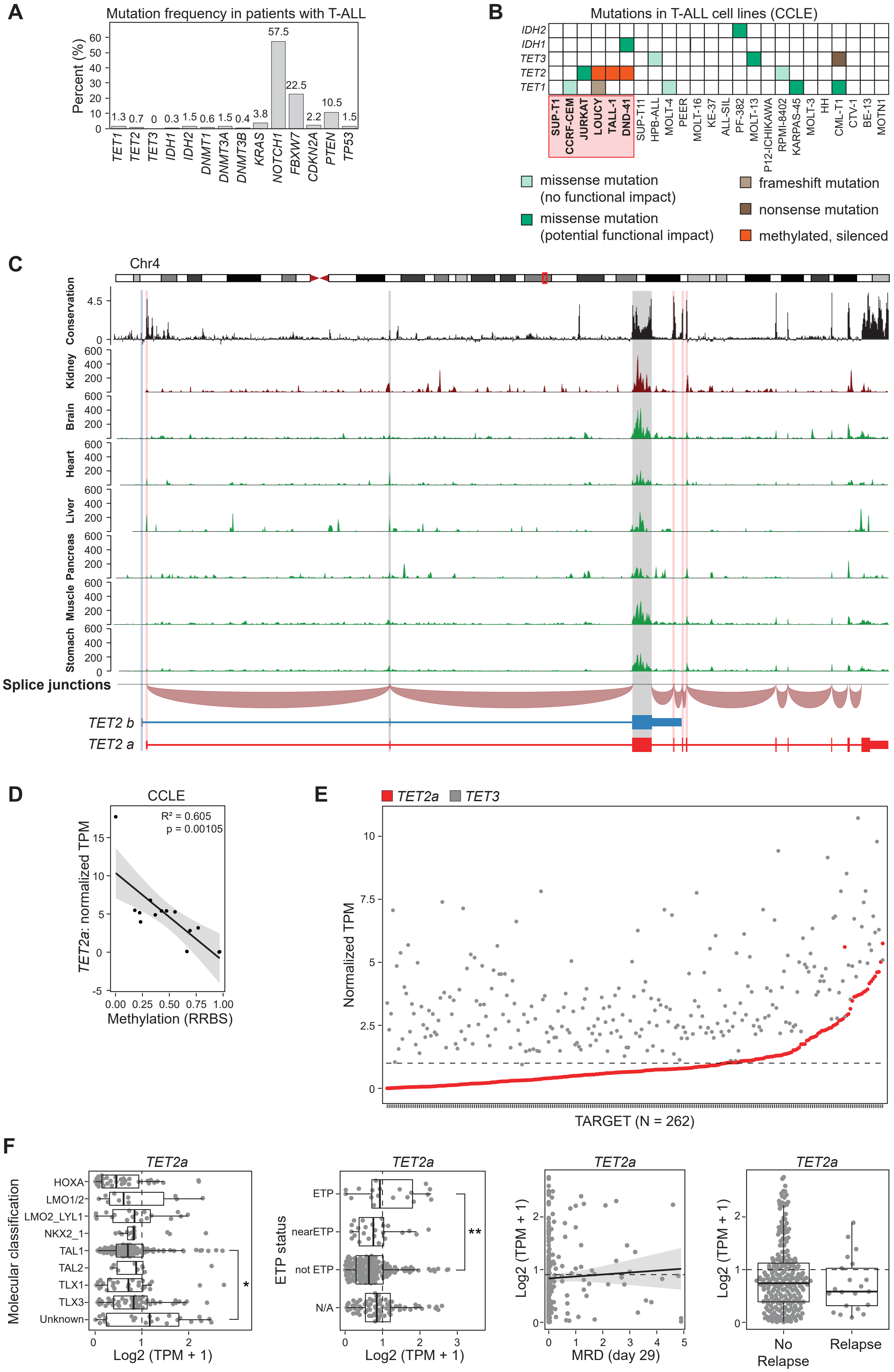

Bensberg et al - Supplementary Figure 2

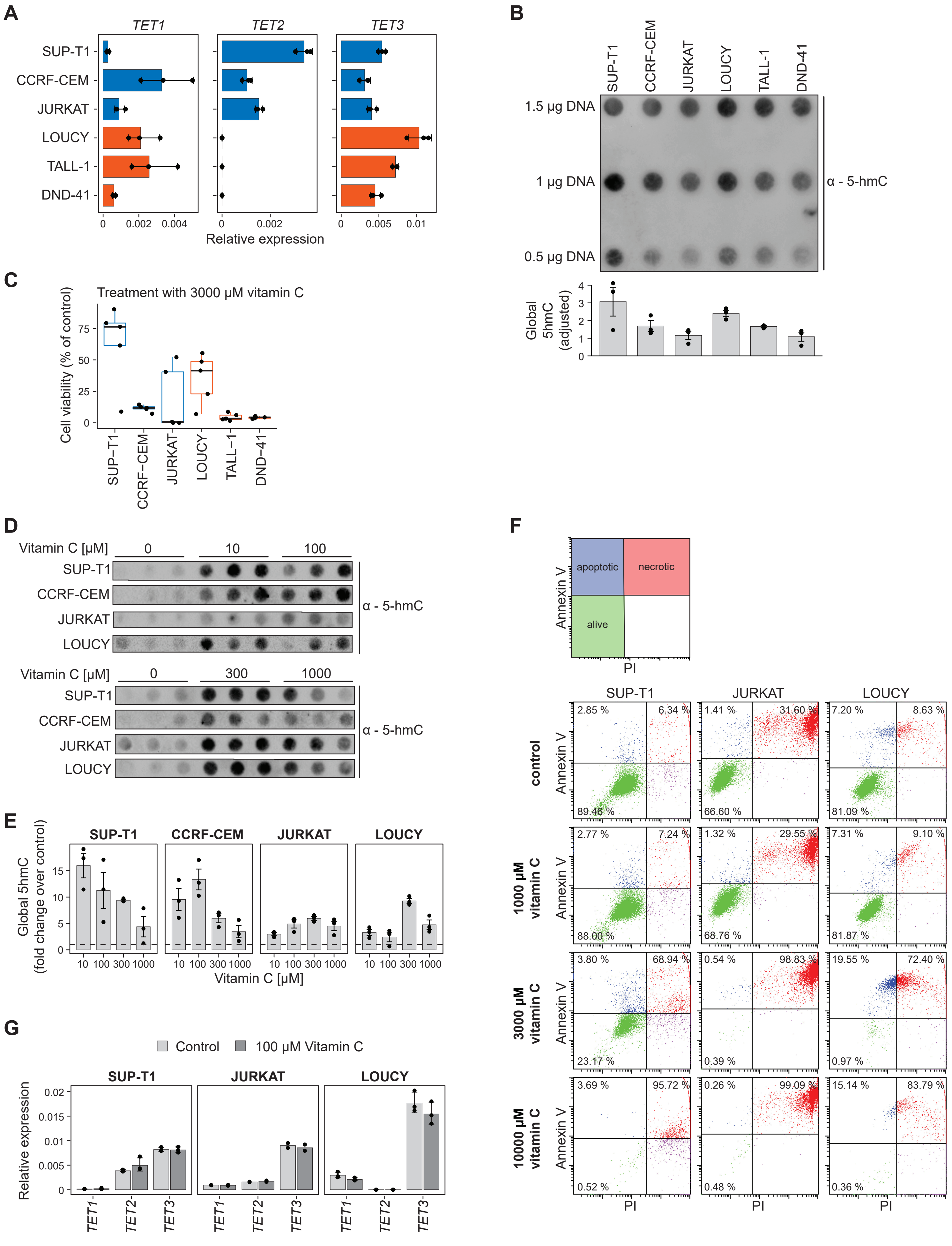

Bensberg et al - Supplementary Figure 3

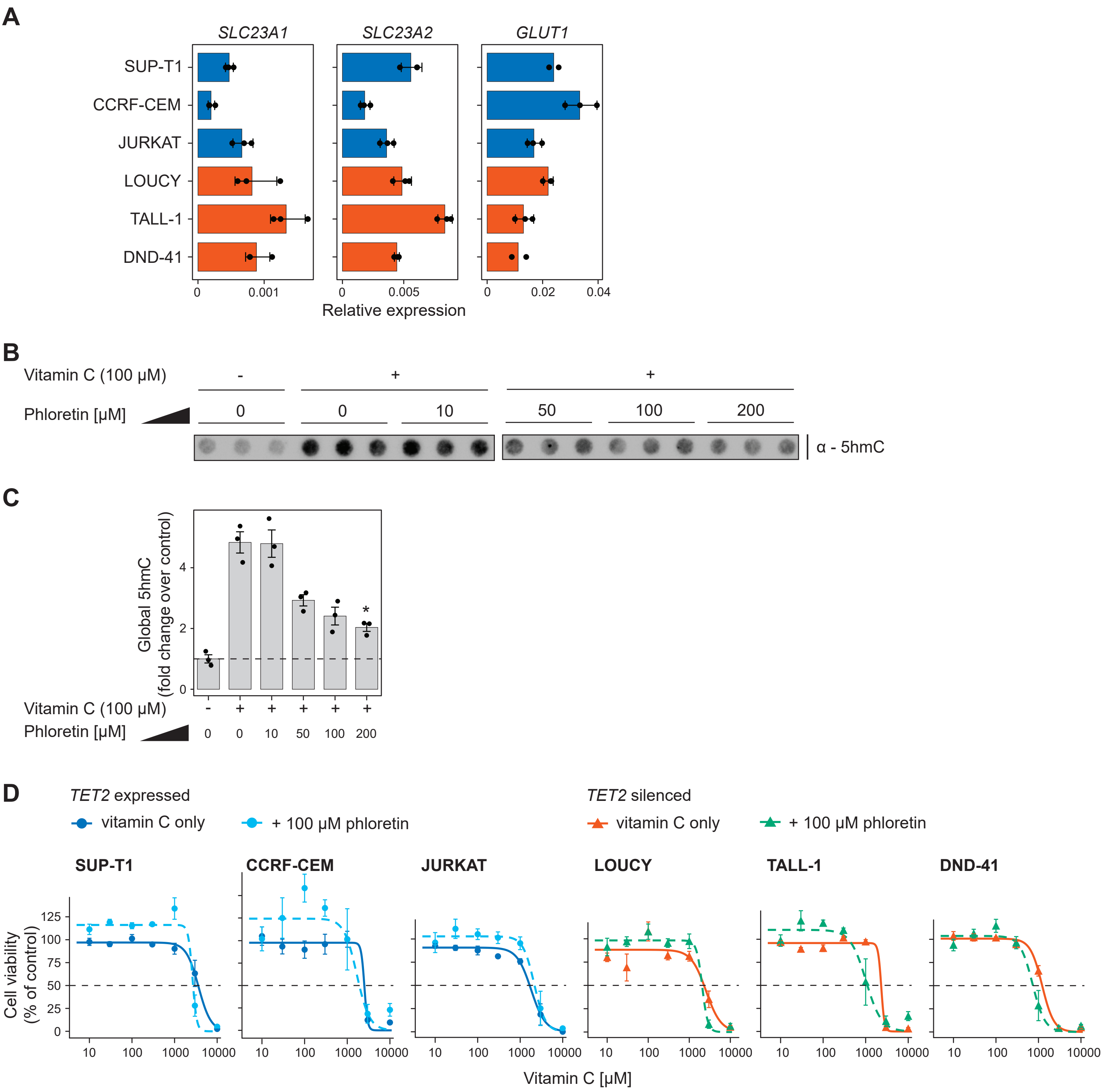

Bensberg et al - Supplementary Figure 4

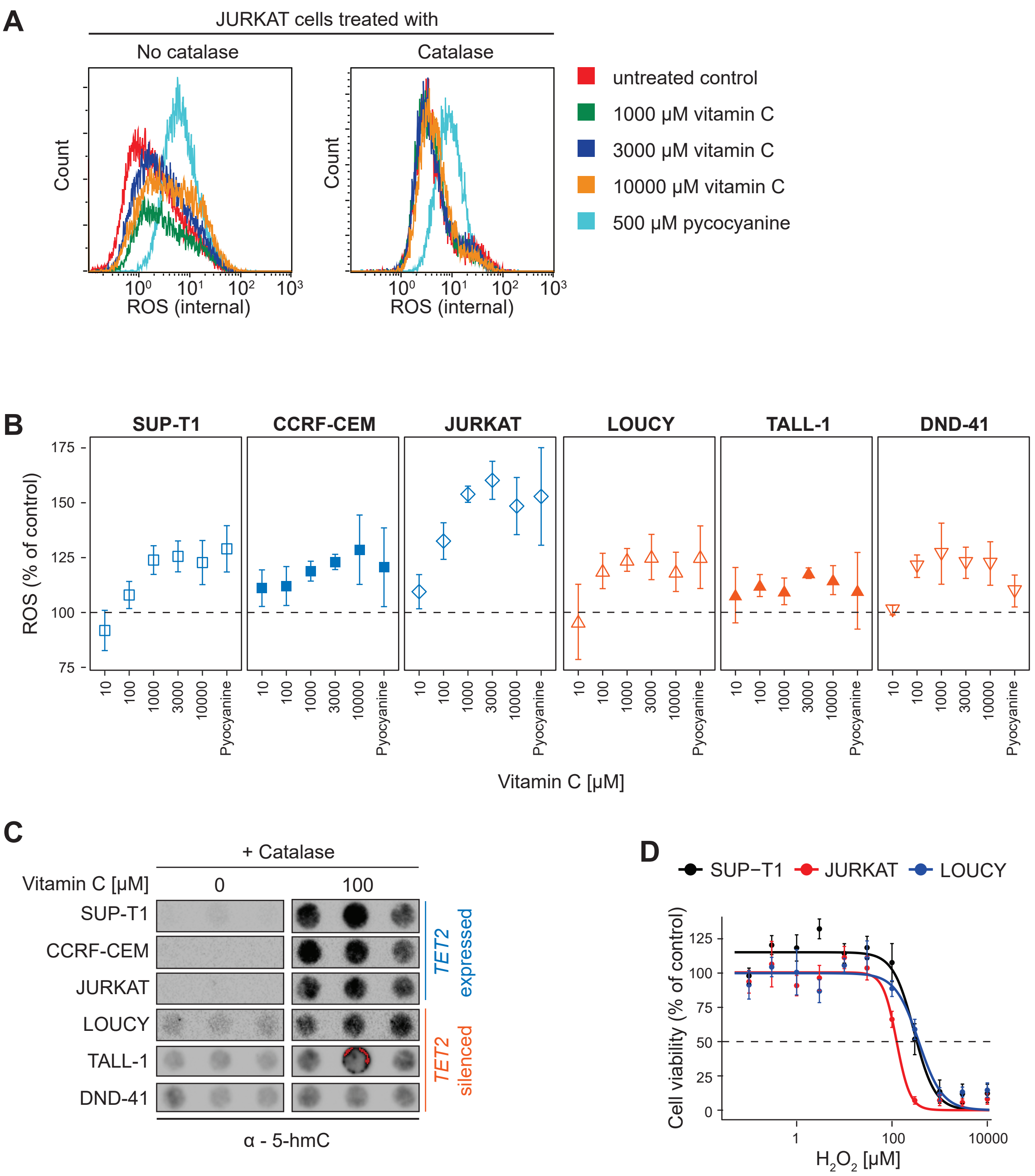

Bensberg et al - Supplementary Figure 5

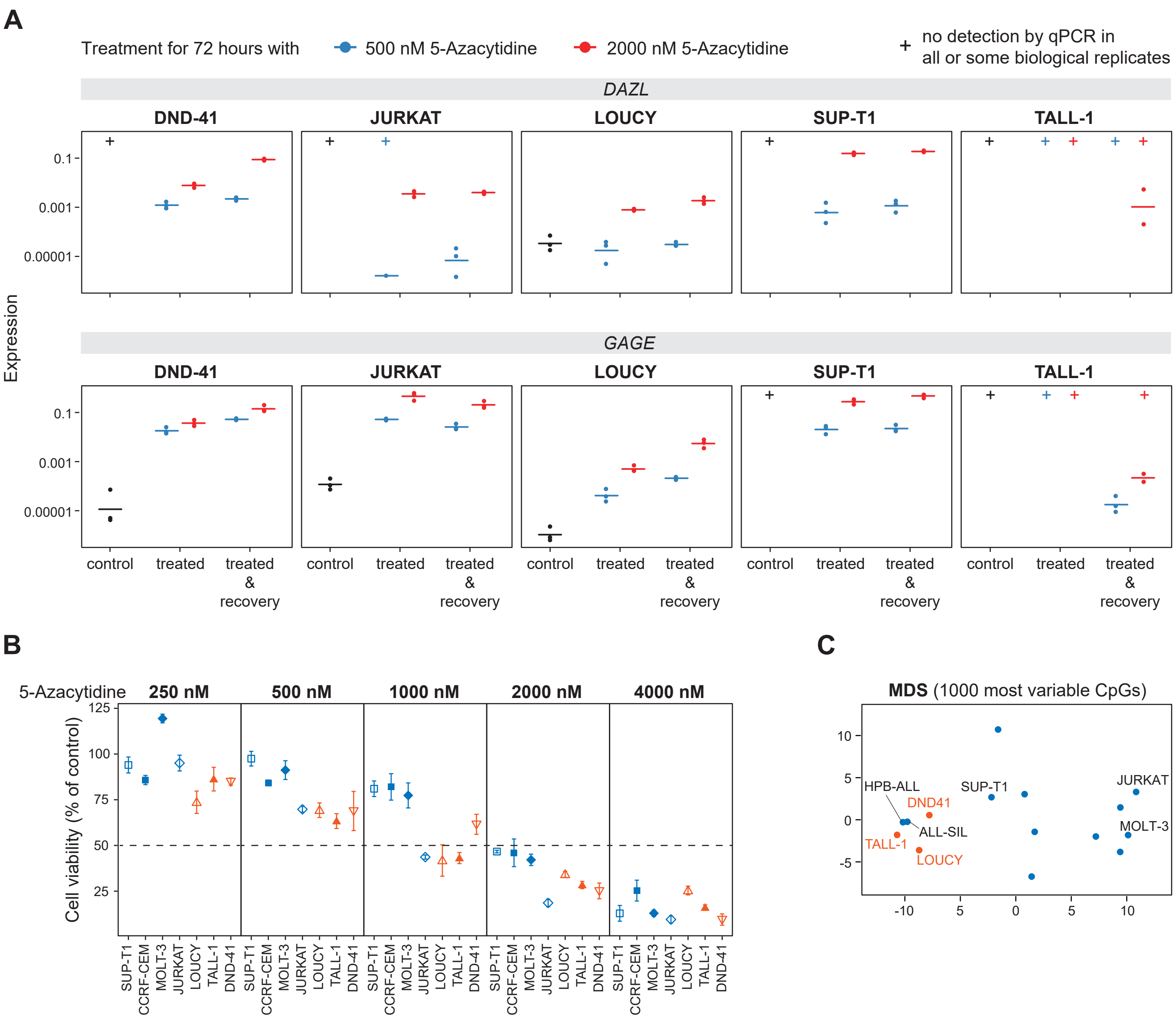

Bensberg et al - Supplementary Figure 6

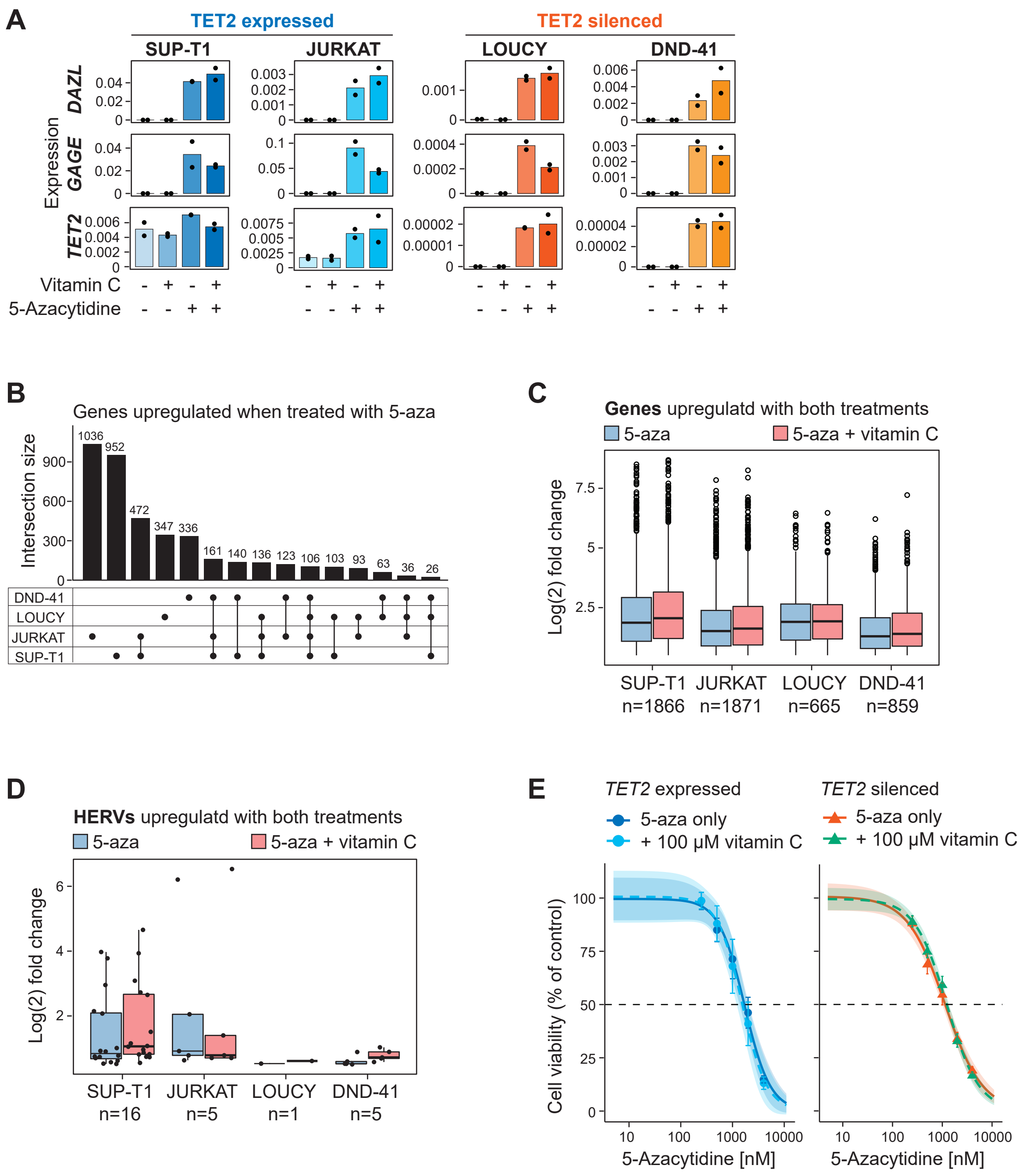

SUPPLEMENTARY FIGURE LEGENDS

Supplementary Figure S1: (**A**) Mutation frequency in genes involved in DNA methylation and known oncogenes in primary T-ALL patients. (**B**) Mutations in *TETs* and *IDHs* in 24 T-ALL cell lines according to Broad Institute Cancer Cell Line Encyclopedia (CCLE). (**C**) Conservation of *TET2* isoforms based on data from 100 vertebrates from UCSC (phylop100way) (top panel). Splice junctions and reads mapping to *TET2* isoforms in seven fetal tissue samples, including kidney (red). Isoform specific exons are marked in red (*TET2a*) and blue (*TET2b*) while shared exons are indicated in grey. Splice junctions for *TET2a* are indicated in red, no *TET2b* specific splice junctions were detected. (**D**) Linear regression association of expression of *TET2a* with methylation of the *TET2* promoter (1kb upstream of the transcription start site) in T-ALL cell lines, based on data from CCLE. (**E**) Expression of *TET2a* (red) and *TET3* (grey) in primary T-ALL patients from the TARGET cohort. Dotted black line: silenced *TET2* (TPM < 1). (**F**) Association of *TET2a* expression with clinical parameters: molecular classification, ETP status, minimal residual disease (MRD) and relapse. Linear regression used to test for statistical significance with TAL1 and ETP as reference for molecular classification and ETP status, respectively. ns, $P > 0.05$; *, $P \leq 0.05$; **, $P \leq 0.01$; ***, $P \leq 0.001$.

Supplementary Figure S2: (**A**) Expression of *TET* genes relative to housekeeping gene *GAPDH* in T-ALL cell lines based on qPCR (n = 3). (**B**) Dot blot (upper) and quantification of signal intensity (lower) showing global 5hmC in untreated T-ALL cell lines. Global 5hmC levels were normalized to amount of DNA blotted. (**C**) Box plot showing cell viability of six T-ALL cell lines treated with 3,000 µM vitamin C (n = 5) as percentage of an untreated control. (**D-E**) Global levels of 5hmC in T-ALL cell lines after treatment with a H_2O control or indicated concentrations of vitamin C for 24 hours as (**D**) dot blot and (**E**) quantification of dot intensity relative to an untreated control (dotted line). (**F**) Flow cytometry of three T-ALL cell lines treated with indicated concentrations of vitamin C for 24 hours and stained with Annexin V and propidium iodine (PI); Annexin V^-/PI^- cells are considered alive, Annexin V^+/PI^- and Annexin V^+/PI^+ cell considered dead; apoptotic and necrotic, respectively. (**G**) Expression of *TET* genes relative to housekeeping gene *GAPDH* measured by qPCR in T-ALL cell lines

treated with a H_2O control or 100 µM vitamin C for 24 hours (n = 3 for SUP-T1 and LOUCY; n = 2 for JURKAT).

Supplementary Figure S3: (**A**) Expression of genes involved in cellular import of vitamin C, *SLC23A1*, *SLC23A2* and *SLC2A1* (GLUT1), relative to housekeeping gene *GAPDH* in T-ALL cell lines based on qPCR (n = 3). (**B-C**) Global levels of 5hmC in SUP-T1 cells after treatment with H_2O control or 100 µM vitamin C and increasing levels of phloretin for 24 hours shown as (**B**) dot blot and (**C**) quantification of dot intensity as fold change of an untreated control (dotted line). (**D**) Cell viability curves for T-ALL cell lines treated with increasing concentrations of vitamin C with or without 100 µM phloretin as percentage of an untreated control (n ≥ 3 for each cell line).

Supplementary Figure S4: (**A**) Intracellular levels of reactive oxygen species (ROS) in JURKAT cells treated with indicated levels of vitamin C in the absence (left panel, two independent replicates) or presence (right panel, one replicate) of catalase as measured by flow cytometry. (**B**) Total (intracellular and extracellular) reactive oxygen species (ROS) created in response to treatment of T-ALL cell lines with vitamin C at increasing concentrations relative to an untreated control (n = 3 for each cell line). (**C**) Dot blot showing global levels of 5hmC in T-ALL cell lines treated with H_2O control or 100 µM vitamin C with the addition of catalase for 24 hours. (**D**) Cell viability curves for three T-ALL cell lines treated with increasing concentrations of hydrogen peroxide (H_2O_2) for 24 hours (n ≥ 5). (**A-B**) Treatment with 500 µM Pyocyanine used as a positive control for ROS production.

Supplementary Figure S5: (**A**) Expression of control genes *DAZL and GAGE* relative to housekeeping gene *GAPDH* in T-ALL cell lines with expressed (SUP-T1, JURKAT) and silenced (LOUCY, DND-41) *TET2* after treatment with 500 nM or 2,000 nM 5-Azacytidine for 72 hours or after treatment followed by recovery without treatment for 5 days. No Ct value in more than one biological replicate indicated as no detection by qPCR (+). (**B**) Cell viability of T-ALL cell lines relative to an untreated control after treatment with indicated concentrations of 5-Azacytidine for 72 hours. Three biological

replicates for each cell line. (**C**) Multidimensional scaling (MDS) plot including the 1000 most variable CpGs for T-ALL cell lines based on reduced representation bisulfite sequencing from Broad Institute Cancer Cell Line Encyclopedia (CCLE).

Supplementary Figure S6: (**A**) Expression of *TET2* and control genes *DAZL* and *GAGE* measured by qPCR relative to housekeeping gene *GAPDH* in T-ALL cell lines with expressed (SUP-T1, JURKAT) and silenced (LOUCY, DND-41) *TET2* after treatment with 2,000 µM 5-Azacytidine and 100 µM vitamin C as indicated for 72 hour. (**B**) Overlap of significantly upregulated genes after treatment with 2,000 µM 5-aza in four T-ALL cell lines which express *TET2* (SUP-T1, JURKAT) or are *TET2*-deficient (LOUCY, DND-41). (**C**) Expression of genes upregulated by treatment with 2,000 µM 5-aza on its own and 5-aza plus 100 µM vitamin C shown by log(2) fold change relative to an untreated control. Data based on total RNA sequencing of biological duplicates for four T-ALL cell lines (*TET2*-expressing: SUP-T1, JURKAT and *TET2*-silenced: LOUCY, DND-41). Dots indicate outliers defined as > 1.5 interquartile range difference to quartile 1 or 3. Number of genes in the gene set for every cell line as indicated. (**D**) Expression of human endogenous retroviruses (HERVs) upregulated by treatment with 2,000 µM 5-aza on its own and 5-aza plus 100 µM vitamin C shown by log(2) fold change relative to an untreated control. Data based on total RNA sequencing of biological duplicates for four T-ALL cell lines (*TET2*-expressing: SUP-T1, JURKAT and *TET2*-silenced: LOUCY, DND-41). Individual HERVs indicated as dots. Number of HERVs included for each cell line as indicated. (**E**) Cell viability curves for T-ALL cell lines treated with increasing concentrations of 5-aza with or without 100 µM vitamin C for cell lines with expressed *TET2* (left panel, biological triplicates for three different cell lines, n=9) or silenced *TET2* (right panel, biological triplicates for three different cell lines, n=9). Cell treated for 72 hours, with treatment refreshed after 24 hours and 48 hours.

SUPPLEMENTARY DATA

Supplementary Dataset S1: Studies included in meta-analysis of mutations in primary T-ALL patients (Figures 1A and S1A).

Supplementary Dataset S2: Accession numbers for RNA-seq of fetal tissue included in analysis of *TET2* isoforms (Figure S1C).

Supplementary Dataset S3: Details about samples analyzed by total RNA sequencing (Figure 4).

Supplementary Dataset S4: Enrichment test for promoter hypermethylation in untreated control samples for genes upregulated by treatment 2,000 µM 5-Azacytidine and 100 µM vitamin C (methylation > 0.7).

Supplementary Dataset S5: Enrichment test for low expression in untreated control samples for genes upregulated by treatment with 2,000 µM 5-Azacytidine and 100 µM vitamin C (low/no expression: TPM < 1).

Supplementary Dataset S6: List of genes upregulated in both *TET2*-silenced cell lines (LOUCY and DND-41) but not upregulated in either of the *TET2*-expressing cell lines (SUP-T1, JURKAT).

Supplementary Dataset S7: Hypergeometric test for enrichment of cancer testis antigens and tumor suppressor genes for up- and downregulated genes in response to treatment with 2,000 µM 5-Azacytidine and 100 µM vitamin C.

Supplementary Dataset S8: Taq-Man probes used for qPCR.
